# Senescence‐Associated Secretory Phenotype (SASP) and Atherosclerotic Plaque Inflammation: Natural Product‐Based Interventions and Implications for Personalized Therapy

**DOI:** 10.1155/crp/6765242

**Published:** 2026-11-10

**Authors:** Dekun Liu, Napamanee Kornthong, Shuguang Yu, Jakkree Tangthianchaichana, Prasert Sobhon, Sha Yang

**Affiliations:** ^1^ School of Acupuncture Moxibustion and Tuina Chengdu University of Traditional Chinese Medicine Chengdu 611137 China cdutcm.edu.cn; ^2^ Chulabhorn International College of Medicine Thammasat University Khlong Luang District Bangkok Pathum Thani 12120 Thailand tu.ac.th; ^3^ Research Unit in Innovative Marine Biotechnology and Natural Bio-Resources for Sustainable Health and Wellness Thammasat University Bangkok Thailand tu.ac.th; ^4^ Acupuncture and Chronobiology Key Laboratory of Sichuan Province Chengdu 610075 China; ^5^ Key Laboratory of Acupuncture for Senile Disease (Chengdu University of TCM) Chengdu University of Traditional Chinese Medicine Ministry of Education Chengdu 611137 China meb.gov.tr; ^6^ Acupuncture Point Effects Key Laboratory of Sichuan Province Chengdu University of Traditional Chinese Medicine Chengdu 611137 China cdutcm.edu.cn; ^7^ Department of Anatomy Faculty of Science Mahidol University Bangkok Thailand mahidol.ac.th

**Keywords:** atherosclerosis, natural product senotherapeutics, plaque inflammation, senescence-associated secretory phenotype, tetramethylpyrazine

## Abstract

Atherosclerosis underlies most ischemic cardiovascular events, and its prevalence rises with population aging. Cellular senescence is increasingly implicated as a driver of plaque progression, although the causal evidence is largely preclinical. Senescent endothelial cells, smooth muscle cells, and macrophages acquire a senescence‐associated secretory phenotype (SASP), releasing cytokines, chemokines, proteases, and procoagulant factors. These factors sustain plaque inflammation, weaken the fibrous cap, and increase rupture risk. In this narrative review, we link SASP signaling to plaque instability through NF‐κB, p38 MAPK, mTOR, NLRP3, and cGAS–STING signaling. We then evaluate tetramethylpyrazine (ligustrazine), quercetin, fisetin, resveratrol, and curcumin against an operational definition reserving senolytic and senomorphic for effects shown in confirmed senescent cells. On that standard, only quercetin and fisetin qualify; for the other three, the vascular evidence is anti‐inflammatory, antioxidant, or senescence‐preventive rather than directed at established senescent cells. Tetramethylpyrazine protects the endothelial glycocalyx and reduces plaque area in atherosclerotic rabbits. Dasatinib plus quercetin benefits ApoE−/− mice, whereas the only human evidence comes from quercetin monotherapy, which improved endothelium‐dependent vasodilation in vessels from symptomatic male, but not female, bypass patients ex vivo. Because senescence burden varies between individuals, we consider how multiomics profiling might stratify patients. The evidence remains mechanistic and hypothesis‐generating, with limited human cardiovascular interventional data: Much of it was recorded in cells never shown to be senescent, and no formulation has been shown to reach the human vessel wall. Targeting the SASP therefore requires trial evidence rather than an established basis for precision medicine.

## 1. Introduction

Atherosclerosis is the predominant pathological condition of myocardial infarction and ischemic stroke and contributes to a substantial share of global morbidity and mortality [1]. An aging population has compounded this disease burden, as advanced age is the strongest nonmodifiable risk factor for atherosclerotic cardiovascular disease. Cardiovascular risk is now predicted more accurately by biological age, which is determined by the accumulation of cellular dysfunction and tissue degradation, than by chronological age. Cellular senescence, an irreversible cell cycle arrest accompanied by metabolic and secretory reprogramming, sits at the core of this biological aging process and directly drives vascular pathology [2].

Vascular aging involves endothelial dysfunction, phenotypic switching of vascular smooth muscle cells (VSMCs), and chronic activation of inflammatory programs in the arterial wall. These changes create a proatherogenic microenvironment that promotes lipid retention, immune cell infiltration, and loss of extracellular matrix integrity [3]. Senescence is not confined to its originating cell. Senescent cells release cytokines, chemokines, growth factors, proteases, and other bioactive molecules through paracrine signaling, which is primarily influenced by the senescence‐associated secretory phenotype (SASP). These molecules propagate the senescent state to neighboring cells [4].

The SASP is context‐dependent. SASP factors can recruit immune cells to eliminate damaged or transformed cells during the acute phase, such as wound healing or tumor suppression. However, chronic accumulation leads to sterile inflammation, tissue disintegration, and progressive organ dysfunction due to the persistent SASP signaling and senescent cells [5]. Within atherosclerotic plaques, this generates a self‐perpetuating inflammatory milieu: SASP factors activate the endothelium, recruit monocytes, drive foam cell formation, erode the fibrous cap through matrix metalloproteinase (MMP) release, and propagate senescence to surrounding cells. The net effect is plaque destabilization and increased rupture risk [4].

Three vascular cell populations cooperate to translate senescence into plaque vulnerability. Senescent endothelial cells lose antithrombotic and barrier properties while upregulating leukocyte adhesion molecules. Senescent VSMCs produce less collagen, thinning the fibrous cap, and simultaneously secrete SASP factors that maintain local inflammation. Senescent macrophages and foam cells sustain a proinflammatory secretory loop within the lipid‐rich core [6]. Genetic and pharmacological clearance of senescent cells in animal models reduces lesion burden and improves plaque composition, establishing senescence as a tractable therapeutic target [7].

In this narrative review, we examine the biology of vascular senescence and atherosclerosis from a mechanistic and translational standpoint. We describe how stress signals converge on the transcriptional programs that maintain the SASP and then evaluate five natural product‐based interventions against explicit operational criteria for senolytic activity (clearance of confirmed senescent cells) and senomorphic activity (suppression of SASP secretion by confirmed senescent cells), giving particular attention to tetramethylpyrazine (TMP). Due to the significant variability in individual senescence load and SASP composition, we also describe how omics technologies and biomarker advancement could facilitate personalized senotherapy. A clearer understanding of the SASP, as both a catalyst of plaque inflammation and a therapeutic target, provides a basis for targeted therapies in aging‐related atherosclerotic disease.

This article is a narrative, nonsystematic review. Relevant literature was identified primarily through PubMed/MEDLINE, supplemented by Web of Science, covering publications from database inception to June 2026, with the search updated during revision. Search terms were combined from three groups: (i) “cellular senescence,” “senescence‐associated secretory phenotype,” “SASP,” “senolytic,” “senomorphic”; (ii) “atherosclerosis,” “plaque inflammation,” “endothelial cell,” “vascular smooth muscle cell,” “macrophage”; and (iii) “natural product,” “tetramethylpyrazine,” “quercetin,” “resveratrol,” “curcumin,” “fisetin.” English‐language original studies, clinical trials, and reviews were considered, and the reference lists of retrieved articles were screened for additional sources. Article selection was purposive rather than exhaustive, based on mechanistic relevance and, where evidence was conflicting, on preference for primary data. No formal eligibility grid, duplicate screening, PRISMA flow diagram, or risk‐of‐bias assessment was applied, consistent with the narrative design of this review.

## 2. Cellular Senescence and SASP in Atherosclerosis

### 2.1. Senescent Cell Populations in Atherosclerotic Lesions

Diverse populations of senescent cells exist within each atherosclerotic plaque, each possessing a unique SASP signature. When these signatures are combined, they result in the progression of the disease. The endothelial cells at the blood‐vessel wall interface are subjected to a constant hemodynamic strain, oxidized lipids, inflammatory mediators, and reactive oxygen species (ROS). These stressors induce senescence mechanisms in these cells by inducing DNA damage, telomere loss, and mitochondrial impairment [8, 9]. Consequently, these cells exhibit elevated levels of p16INK4a and p21CIP1. Impaired nitric oxide synthesis, increased vascular permeability, and overexpression of adhesion molecules (e.g., VCAM‐1 and ICAM‐1) that facilitate monocyte adhesion and transmigration into the intima are the defining characteristics of this endothelial cell senescence [10].

The senescence of VSMCs in atherosclerotic lesions also occurs through various processes, such as oxidative stress, inflammatory mediators, DNA damage, and telomere dysfunction, which occur during proliferative expansion. Senescent VSMCs lose their contractile ability and show reduced capacity to produce collagen, which is essential for sustaining the integrity of the fibrous cap. At the same time, these cells upregulate MMPs, including MMP‐2, MMP‐9, and MMP‐13, that degrade extracellular matrix components and destabilize the plaque [11]. Interestingly, the topographical distribution of senescent VSMCs usually correlates with cap thinning and high‐risk rupture‐prone areas.

Macrophages, along with their lipid‐ingested derivatives (foam cells), comprise another significant senescent population in plaques. Macrophage senescence—a growth‐arrested, yet viable and metabolically active state induced by chronic exposure to oxidized low‐density lipoprotein (LDL) (ox‐LDL), cholesterol crystals, lysosomal stress, and inflammatory signals—is a phenomenon regulated by early warning signals through an integrated set of signaling pathways. In contrast to replicative senescence in other cell types, macrophage senescence is often independent of telomere shortening and is largely induced by lipotoxic stress and prolonged inflammatory signaling [6]. Senescent macrophages demonstrate impaired phagocytic capacity, defective efferocytosis (clearance of dying cells), and persistent release of proinflammatory cytokines, consequently driving inflammation of the necrotic core and chronic inflammation within the plaque [7].

### 2.2. SASP Composition in the Vascular Context

The SASP includes a complex and heterogeneous array of secreted factors, with its composition varying depending on cell type, senescence inducer, and tissue microenvironment. Within atherosclerotic lesions, the SASP exhibits modular sets of paracrine molecules that include inflammatory cytokines (e.g., interleukin‐1β [IL‐1β], IL‐6, TNF‐α), chemokines (e.g., CCL2/monocyte chemoattractant protein‐1 [MCP‐1], CXCL8/IL‐8, CXCL1), matrix‐remodeling enzymes (e.g., MMP‐2, MMP‐9, MMP‐12, ADAMTS family members), procoagulant factors (e.g., plasminogen activator inhibitor‐1 [PAI‐1], tissue factor), growth factors (e.g., VEGF, TGF‐β), and lipid mediators [4, 12]. Recent profiling studies utilizing multiplexed protein arrays and secretome proteomics have revealed that individual SASP signatures can contain dozens to hundreds of secreted proteins, underscoring the profound secretory transformation that accompanies senescence.

The variations in SASP composition associated with cell‐type differences indicate differences in cellular activities and exposures to stress. Senescent endothelial cells favor the release of adhesion‐enhancing factors and chemokines that recruit immune cells, as well as excessive amounts of PAI‐1, which promotes thrombosis. Senescent VSMCs generate elevated concentrations of MMPs and, at the same time, show decreased collagen and elastin synthesis, which directly compromises vascular structure. Senescent macrophages maintain high secretion of the cytokines IL‐1β, IL‐6, and TNF‐α, which form a positive feedback loop that maintains local inflammation and promotes paracrine senescence in adjacent cells [13, 14].

### 2.3. Consequences for Plaque Stability

The cumulative impact of SASP factors on atherosclerotic plaques has a significant effect on lesion composition and stability. The SASP of senescent endothelial cells enhances sustained immune cell recruitment, which maintains inflammatory infiltration and fuels atherosclerotic plaque progression. The chemokines involved in this process include CCL2 and CXCL8, which form concentration gradients that drive monocyte migration from the circulation into the vasculature, whereas increased adhesion molecules promote robust adhesion and transmigration. In the intima, recruited monocytes differentiate into macrophages through the action of local cytokines, resulting in a cycle that increases the population of inflammatory cells [15].

One of the determinants of plaque vulnerability is matrix degradation, a critical SASP‐mediated process. Senescent VSMCs and macrophages produce MMPs that actively break down collagen fibers, which weakens the fibrous cap. Concurrently, cap repair and reinforcement are impaired due to the reduced collagen synthesis capacity of senescent VSMCs. This imbalance between degradation and synthesis causes progressive erosion of the cap, thus predisposing the plaque to mechanical rupture during hemodynamic strain. Plaques with thin fibrous caps that overlie large necrotic cores are known as thin‐cap fibroatheromas and are the morphological subtype most likely to rupture and cause thrombotic complications [16].

Paracrine senescence transmission further amplifies the local senescence burden beyond the initial senescent cell population. SASP factors, including IL‐1α, IL‐6, IL‐8, and ROS, can induce senescence in adjacent healthy cells through mechanisms involving DNA damage, mitochondrial dysfunction, and sustained activation of stress signaling pathways [17]. This process generates expanding zones of senescent cells within plaques, particularly in regions subjected to heightened mechanical stress or inflammation. Direct evidence that these zones map onto the plaque shoulders and sites of cap disruption that carry the highest rupture risk is not yet available for human lesions, because spatially resolved transcriptomics of vascular senescence has so far been performed in mice [18]; the human data remain histological, showing senescence markers and markedly shortened telomeres in fibrous‐cap smooth muscle cells [19].

### 2.4. Evidence From Senescent Cell Clearance Studies

Experimental validation of senescence as a causative driver of atherosclerosis comes from genetic and pharmacological studies targeting senescent cells. Research employing INK‐ATTAC mice, which express a suicide gene under the control of the p16^INK4a^ promoter, has demonstrated that selective elimination of p16‐expressing cells reduces atherosclerotic burden and improves plaque composition in LDL receptor‐deficient (LDLR^−/−^) mice. The clearance of senescent cells decreases the expression of inflammatory markers, reduces necrotic core size, and increases fibrous‐cap thickness, collectively indicating enhanced plaque stability [7]. Pharmacological senolytic interventions, such as the combination of dasatinib and quercetin, similarly reduce senescent cell burden and improve vascular function in both aged and atherosclerotic mouse models, although in atherosclerotic mice, this clearance is restricted to the medial layer and does not reduce lesion size. These findings support the modulation of the SASP as a viable therapeutic strategy for atherosclerosis [20].

## 3. Molecular Drivers of the SASP in Atherosclerotic Cells

### 3.1. DNA Damage Response (DDR) and Genomic Instability

Persistent DNA damage serves as a fundamental trigger and perpetuating factor for both cellular senescence and the SASP. Within atherosclerotic lesions, vascular cells are subjected to a multitude of genotoxic stresses, such as oxidized lipids, inflammatory mediators, advanced glycation end products, and intrinsic replication stress associated with proliferative responses. These stressors induce DNA double‐strand breaks, DNA modifications, and telomere dysfunction, which in turn activate the DDR machinery centered on the ATM and ATR kinases. In contrast to a transient DDR that enables repair, persistent DDR signaling establishes a stable senescent state. This state is characterized by the sustained expression of p53 and p21^CIP1^, which enforce irreversible cell cycle arrest [21].

The DDR is directly linked to the transcriptional programming of the SASP through several interconnected mechanisms. A primary connection is the DNA damage‐induced activation of nuclear factor‐κB (NF‐κB), wherein ATM‐mediated signaling facilitates the nuclear translocation of NF‐κB subunits, thereby driving the transcription of inflammatory genes. Concurrently, DDR signaling leads to the suppression of Lamin B1, resulting in chromatin reorganization that renders previously silent inflammatory genes accessible for activation. Furthermore, the release of cytoplasmic and mitochondrial DNA fragments during genomic instability activates the cGAS–STING pathway, adding another layer of inflammatory drive [22]. These pathways convert an acute stress response into the chronic, proinflammatory secretory program that defines the SASP.

### 3.2. NF‐κB: The Master SASP Transcriptional Switch

NF‐κB stands as the principal transcriptional regulator of the SASP, coordinating the expression of a vast array of inflammatory secreted factors. In nonsenescent cells, NF‐κB dimers are typically sequestered in the cytoplasm through binding to inhibitory IκB proteins. Various senescence‐inducing stimuli, including DNA damage, oxidative stress, inflammatory cytokines, and telomere dysfunction, converge to activate the IκB kinase (IKK) complex. IKK phosphorylates IκBα, targeting it for proteasomal degradation and thereby releasing NF‐κB for translocation into the nucleus. Once in the nucleus, NF‐κB binds to κB response elements within the promoters of target genes, directly driving the transcription of key SASP components such as IL‐1α, IL‐1β, IL‐6, IL‐8, and CCL2 [23].

The significance of NF‐κB in maintaining the SASP is further illustrated by autocrine amplification loops. A prominent example involves IL‐1α, which can be presented on the cell membrane or released upon cell damage. This cytokine engages the IL‐1 receptor on the senescent cell itself, initiating a positive feedback loop that perpetuates NF‐κB activation and SASP secretion. Importantly, experimental inhibition of NF‐κB significantly reduces the expression of SASP factors without reversing the growth arrest, indicating that NF‐κB specifically regulates the secretory phenotype rather than the initiation of senescence itself. Within the context of atherosclerosis, heightened NF‐κB activity in senescent cells amplifies local inflammatory signaling and actively contributes to plaque progression [13].

### 3.3. p38 Mitogen‐Activated Protein Kinase (MAPK) and Stress Response Amplification

The p38 MAPK pathway acts as a critical stress sensor and amplifier of the SASP in senescent vascular cells. Diverse stressors relevant to atherosclerosis, including oxidative stress, inflammatory cytokines, and DNA damage, converge to activate p38 via upstream kinase cascades. Activated p38 then phosphorylates various transcription factors, such as ATF2 and CHOP, and modulates components of the NF‐κB pathway, thereby enhancing their transcriptional activity. Beyond transcription factor regulation, p38 also post‐transcriptionally stabilizes SASP mRNA transcripts through interactions with specific RNA‐binding proteins. This dual action increases the translational efficiency and subsequent secretion of inflammatory factors [24].

In the atherosclerotic lesion microenvironment, p38 activation forms a feed‐forward loop that sustains the SASP. For instance, exposure to ox‐LDL and inflammatory signals activates p38, which in turn promotes the secretion of factors that further exacerbate stress. Experimental inhibition of p38 has been shown to reduce the expression of multiple SASP components, including IL‐6, IL‐8, and various MMPs, in senescent endothelial cells and macrophages. The p38 pathway appears to be particularly crucial for stress‐induced senescence, as opposed to replicative senescence, positioning it as a key mechanistic node that links the pathological microenvironment of atherosclerosis to the persistent inflammatory output of senescent cells [24].

### 3.4. Mechanistic Target of Rapamycin (mTOR) and SASP Protein Synthesis

The mTOR pathway, a central regulator of protein synthesis, is indispensable for maintaining the high secretory output characteristic of the SASP. Despite being in a state of growth arrest, senescent cells exhibit elevated metabolic activity and biosynthetic capacity, with the production of SASP factors constituting a significant anabolic burden. mTOR Complex 1 (mTORC1) promotes the translation of SASP components by phosphorylating key downstream effectors, including the translational repressor 4E‐BP1 and S6 kinase. Studies demonstrate that pharmacological inhibition of mTOR with rapamycin or its analogs effectively reduces the secretion of SASP factors without inducing senescent cell death or altering canonical senescence markers. This defines mTOR primarily as a target for senomorphic (SASP‐suppressing) rather than senolytic (cell‐clearing) interventions [25].

The mTOR pathway is involved in wide crosstalk with other SASP regulatory networks; mTOR activity may enhance NF‐κB signaling, and the mRNAs produced by NF‐κB target genes then need mTOR‐dependent translation. mTOR is also involved in the regulation of autophagy. The reduction of autophagic flux normally related to senescent cells results in the accumulation of damaged organelles themselves capable of continuing to signal inflammatory reactions. In atherosclerotic plaques, the senescence‐specific activation of mTOR in the senescent cells of the atherosclerotic vessel environment also supports the long‐term production of SASP, which implies that mTOR inhibitors might be therapeutically effective in inhibiting this negative secretory phenotype [26].

### 3.5. Additional SASP Regulatory Pathways

Beyond the core pathways involving NF‐κB, p38 MAPK, and mTOR, several other mechanisms significantly contribute to the regulation and maintenance of the SASP. The NLRP3 inflammasome, activated by damage‐associated molecular patterns and crystalline cholesterol, plays a specialized role. It activates caspase‐1, which cleaves pro‐IL‐1β into its mature, secreted form, and inflammasome‐mediated IL‐1 signaling in turn controls expression of the wider secretory program and its capacity to transmit senescence to neighboring cells [17]. This mechanism is especially pertinent in senescent macrophages within plaques, which are directly exposed to cholesterol crystals. Simultaneously, the cGAS–STING pathway senses mislocalized cytosolic DNA, triggering Type I interferon responses and ancillary inflammatory signaling [22]. At an epigenetic level, remodeling through histone modifications and changes in DNA methylation creates a permissive chromatin environment at inflammatory gene loci, facilitating their sustained transcription. These diverse yet interconnected pathways collectively establish and maintain the robust inflammatory program of the SASP, thereby revealing multiple potential points for therapeutic intervention [5]. These mechanisms are summarized in Figure 1.

**FIGURE 1:**
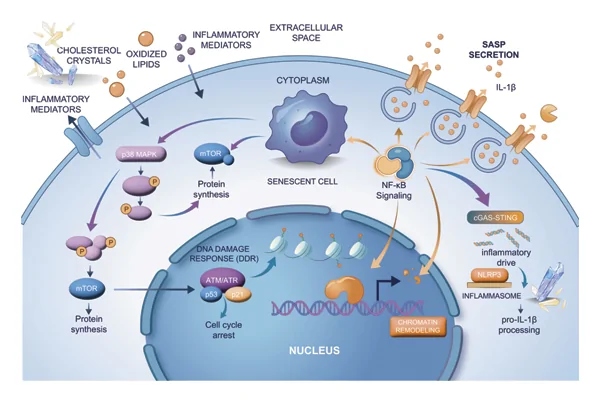
Molecular mechanisms driving the SASP in senescent vascular cells.

Schematic of a single senescent vascular cell (endothelial cell, VSMC, or macrophage) with the plasma membrane, cytoplasm, mitochondrion, and nucleus shown as nested compartments. Atherogenic stressors at the cell surface—ox‐LDL, cholesterol crystals, ROS, and mechanical strain—activate four parallel intracellular axes (DDR, p38 MAPK, mTOR, and NF‐κB) whose outputs converge on the nucleus, where chromatin remodeling renders SASP gene loci accessible to sustained transcription. Intracellular danger sensors NLRP3 (caspase‐1/IL‐1β maturation) and cGAS–STING (cytosolic DNA sensing) feed forward into the same network. The integrated transcriptional output is depicted as SASP secretion of cytokines, chemokines, and matrix‐degrading enzymes via vesicles into the extracellular space, where they propagate paracrine senescence and inflammation to neighboring vascular cells, sustaining plaque instability. Mechanistic detail for each pathway is presented in Section 3. Abbreviations: DDR, DNA damage response; ox‐LDL, oxidized low‐density lipoprotein; ROS, reactive oxygen species; SASP, senescence‐associated secretory phenotype; VSMC, vascular smooth muscle cell.

## 4. Proinflammatory SASP Factors and Their Effects on Atherosclerotic Plaques

### 4.1. Inflammatory Cytokines and Endothelial Dysfunction

One of the typical SASP cytokines is IL‐6, which has widespread implications for vascular pathophysiology. The senescent endothelial cells, VSMCs, and macrophages found in the plaque secrete this cytokine at high levels [4, 14]. Locally, IL‐6 enhances the expression of endothelial adhesion molecules, increases vascular permeability, and polarizes immune cells to adopt proinflammatory phenotypes. It interacts with the IL‐6 receptor/gp130 complex to mediate its signaling, leading to the activation of the JAK–STAT pathway. This not only promotes the production of acute phase proteins but also supports chronic inflammation. In the environment of an atherosclerotic plaque, the effects of IL‐6 are directly involved in the main pathogenic mechanisms, i.e., monocyte recruitment, foam cell development, and extracellular matrix remodeling [27].

Another key SASP molecule is IL‐1β, whose expression is notably high in senescent macrophages, in which activation of the NLRP3 inflammasome promotes its maturation and release. IL‐1β triggers endothelial cells to increase the expression of adhesion molecules VCAM‐1, ICAM‐1, and E‐selectin to promote the adhesion and transmigration of leukocytes. In addition to the endothelium, IL‐1β promotes VSMC proliferation and migration and increases the expression of matrix‐degrading enzymes. One of the crucial elements of IL‐1β action is the ability of this cytokine to stimulate NF‐κB signaling in its target cells. This not only increases the spread of inflammatory cascades but also induces a senescent state in adjacent cells via paracrine signals, increasing the senescence burden in the area. The clinical importance of this pathway is strongly supported by trials such as CANTOS, where inhibition of IL‐1β with canakinumab induced a decline in recurrent cardiovascular events in patients who had previously experienced a heart attack [28]. Trials of this kind established residual vascular inflammation, which persists in many patients despite optimal lipid lowering, as a treatable target, and biologic agents directed at IL‐1β, IL‐6, IL‐17A, and CD20‐positive B cells are now under evaluation for the same purpose [29]. None of these agents has been tested against a senescence‐specific endpoint in atherosclerosis, so how much of any benefit reflects suppression of SASP output rather than inflammation of other origin remains unresolved.

### 4.2. Chemokines and Immune Cell Recruitment

MCP‐1 or CCL2 is also an essential component of immune cell recruitment in the inflammatory response that drives atherosclerotic lesion formation. It also creates a chemotactic gradient through which circulating monocytes enter the endothelial layer and migrate into the intimal space. VSMCs and senescent endothelial cells contribute to the sustained release of CCL2, which supports a continuous influx of monocytes even in mature, advanced plaques. After entering the vessel wall, these monocytes differentiate into macrophages under the influence of local cues such as macrophage colony‐stimulating factor (M‐CSF). They take up modified lipoproteins and transform into lipid‐laden foam cells that characterize the atherosclerotic core [30].

CXCL8, or IL‐8, acts by attracting neutrophils and subsets of monocytes via receptors CXCR1 and CXCR2. Although neutrophils are not a dominant cell type in stable plaques, their recruitment is massive during acute plaque destabilization, during which they play a role in increasing inflammatory levels and predisposing the plaque to thrombosis. CXCL8 also has direct effects on endothelial cells that promote angiogenesis and vascular permeability. The chemotactic environment of the plaque is also influenced by other SASP‐secreted chemokines such as CXCL1, CXCL5, and CX3CL1.

Together, this ensemble of chemokines creates a potent and sustained chemotactic signal that drives the continuous recruitment of inflammatory cells throughout all stages of plaque development and progression, as illustrated in Figure 2 [15].

**FIGURE 2:**
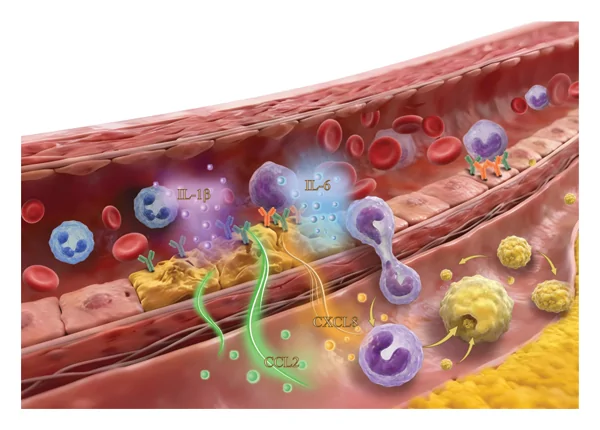
SASP‐mediated immune recruitment and endothelial dysfunction.

Figure 2 illustrates the process of immune cell recruitment and endothelial dysfunction mediated by the SASP. The image depicts the vascular endothelial cell layer, with blood cells such as red blood cells present in the bloodstream. Endothelial cells or related cells release cytokines such as IL‐1β, IL‐6, CXCL8, and CCL2, which are involved in the recruitment of immune cells (e.g., neutrophils and monocytes) to the vascular area. Additionally, the deposition of LDL in the vascular wall is associated with the development of endothelial dysfunction. Overall, the figure demonstrates the pathological process by which SASP recruits immune cells through cytokines and induces endothelial dysfunction.

### 4.3. MMPs and Plaque Destabilization

MMPs constitute a class of SASP factors with a direct and potent impact on the structural integrity of atherosclerotic plaques. MMP‐9, or gelatinase B, degrades denatured collagen and other key matrix components, progressively weakening the protective fibrous cap. Both senescent VSMCs and macrophages secrete elevated levels of MMP‐9, generating a proteolytic microenvironment that erodes the barrier between the thrombogenic lipid core and the circulating blood. Similarly, MMP‐2 (gelatinase A) targets basement membrane constituents and elastin fibers, compromising the fundamental architecture of the vessel wall and further destabilizing the lesion [31].

Other MMPs also contribute significantly to this degradative process. MMP‐12, known as macrophage elastase, plays a specialized role in the breakdown of elastic fibers, while MMP‐13 (collagenase‐3) targets interstitial collagens. Beyond the classic MMPs, members of the ADAMTS family, such as ADAMTS‐4 and ADAMTS‐5, degrade proteoglycans within the extracellular matrix. This aggressive proteolytic activity is compounded by a relative deficiency in their natural inhibitors, the tissue inhibitors of metalloproteinases (TIMPs), within the SASP secretome. This shift in the protease–antiprotease balance decisively tilts the equilibrium toward net matrix degradation. The critical nature of this imbalance in determining plaque vulnerability is underscored by experimental studies in animal models, where pharmacological inhibition of MMPs has been shown to reduce the incidence of plaque rupture [32].

### 4.4. Procoagulant and Prothrombotic Factors

PAI‐1 emerges as a key SASP factor that mechanistically links cellular senescence to an increased risk of thrombosis. Senescent endothelial cells and perivascular adipocytes secrete substantial amounts of PAI‐1. This serpin acts as a potent inhibitor of both tissue‐type plasminogen activator (tPA) and urokinase‐type PA (uPA), effectively suppressing the body’s fibrinolytic capacity. The resultant prothrombotic state is characterized by the more facile formation of fibrin clots and a pronounced resistance to their physiological dissolution. Within atherosclerotic plaques, localized elevations of PAI‐1, particularly in areas enriched with senescent endothelial cells, are posited to play a crucial role in the transition from a vulnerable plaque to an occlusive thrombus following plaque rupture, thereby directly linking senescence biology to acute clinical events [33].

## 5. Natural Product‐Based Senotherapeutics: Mechanisms and Targets

### 5.1. Senotherapeutic Strategies: Overview

Two broad therapeutic strategies target cellular senescence and the SASP, and they are largely complementary. Senolytic agents kill senescent cells, reducing their abundance and the bulk SASP output. Senescent cells survive accumulated damage by upregulating antiapoptotic BCL‐2 family members (BCL‐2, BCL‐XL, BCL‐W), and senolytics exploit this dependence to trigger apoptosis [34]. In contrast, senomorphic agents leave senescent cells in place but silence the pathways that produce and maintain the SASP [35]. Throughout this review, these terms are applied operationally. A compound is called senolytic only where selective loss of viability has been demonstrated in cells first confirmed to be senescent, with the viability of nonsenescent control cells assayed in parallel. It is called senomorphic only where SASP output, measured as secreted protein rather than as intracellular transcript or protein expression, has been reduced in cells first confirmed to be senescent and without killing them. Inhibition of NF‐κB, reduction of ROS, or preservation of endothelial function in cells that were never shown to be senescent is described here as general anti‐inflammatory, antioxidant, or vascular protective activity, and prevention of entry into senescence is kept distinct from action on already established senescent cells.

Both strategies are relevant to atherosclerosis. Senolytics could reduce the senescent cell pool in plaques, suppress chronic SASP release, limit matrix degradation, and improve plaque stability, but abrupt clearance of senescent cells in structural plaque regions could temporarily destabilize the lesion. In principle, senomorphic interventions sidestep this risk by silencing the harmful secretory output while preserving any beneficial functions of the senescent cell (for example, contact‐inhibition signals to neighboring cells). Natural products are unusually well suited to either strategy because individual compounds frequently engage multiple targets and several display dual senolytic and senomorphic activity [36].

These labels carry evidentiary requirements that are easily blurred. To call a compound senolytic, a study must first establish that the target cells are senescent, by senescence‐associated β‐galactosidase (SA‐β‐gal) activity, elevated p16INK4a or p21CIP1, telomere‐associated foci, and stable growth arrest, and must then show preferential loss of those cells, while the viability of nonsenescent controls assayed in parallel is preserved. To call a compound senomorphic, a study must show a measured fall in SASP output, preferably secreted protein in conditioned medium, from cells confirmed senescent before treatment, and without a fall in their number. Neither label is established by NF‐κB inhibition, lower ROS, reduced adhesion molecule expression, or improved vasorelaxation in cells that were never shown to be senescent; such findings define a vascular protective or anti‐inflammatory agent. Preventing entry into senescence is a third, separate activity, and evidence obtained in one tissue does not transfer automatically to the vessel wall. We apply these criteria throughout Section 5. Table 1 summarizes the sources, targets, cardiovascular evidence, and translational limitations of the natural products discussed below.

**TABLE 1:** Natural product senotherapeutics evaluated for atherosclerotic cardiovascular disease.

Compound	Source	Class	Primary targets	Key SASP factors modulated	Cardiovascular preclinical effects	Clinical translation	Bioavailability constraint	Refs
Tetramethylpyrazine (TMP, ligustrazine)	Ligusticum chuanxiong Hort.	Not established as senomorphic; vascular protective, anti‐inflammatory, and antioxidant (no senescence‐specific endpoint has been measured in any vascular cell or aortic tissue; the only confirmed senescent cells tested were nonvascular bone marrow mesenchymal stem/progenitor cells)	NF‐κB; p38 MAPK; TLR4; heparanase‐1	None measured in confirmed senescent cells of any tissue; IL‐6, IL‐8, adhesion molecules, and ROS reduced in nonsenescent stressed endothelial cells	Reduced plaque area in cholesterol‐fed rabbits [37] and in high‐fat‐diet ApoE−/− mice [38], confirmed in a meta‐analysis of 12 studies and 258 animals [39]; protected endothelial glycocalyx in LPS‐stimulated endothelium [40]; attenuated ox‐LDL‐induced endothelial dysfunction; no senescence‐specific endpoint measured in any vascular or atherosclerosis experiment	Clinically used in China for stroke and cardiovascular disease as injectable; no atherosclerosis‐specific trial reported	Short plasma half‐life; extensive first‐pass metabolism; injectable formulations preferred over oral	[37–46]
Quercetin	Apples, onions, berries (dietary flavonol)	Senolytic (selective killing of confirmed senescent human endothelial cells by quercetin alone, with nonsenescent controls assayed in parallel); senomorphic action not demonstrated in confirmed senescent vascular cells	PI3K–AKT prosurvival pathway; BCL‐2/BCL‐XL; AMPK/mTOR; NF‐κB; p16/p21; p53/SERPINE1	IL‐6, IL‐8; MMPs	Dasatinib + quercetin (D + Q) in Western diet‐fed ApoE−/− mice cleared senescent (TAF+) cells from the aortic media but not from within intimal plaques; intimal plaque size, plaque lipid content, macrophage (F4/80) burden and plaque fibrosis were unchanged, whereas intimal plaque calcification and osteogenic (Osterix) signaling were significantly reduced and relaxation to NO donors was improved; quercetin monotherapy reduced aortic lesion area by 46% in Apolipoprotein E–deficient mice [47] and attenuated aortic sinus and thoracic aortic lesion size at 64 mg/kg/day [48], with no senescence endpoint measured in either; in the clearest experiment pairing a lesion endpoint with an in vivo senescence‐specific endpoint, quercetin depleted senescent plaque macrophages and suppressed SASP factors through p38 MAPK/p16 inhibition [49]; quercetin monotherapy, not D + Q, also improved endothelium‐dependent relaxation in ex vivo internal thoracic artery segments from male but not female CABG patients (human ex vivo evidence)	Phase I–II D + Q senolytic trials in diabetic kidney disease, idiopathic pulmonary fibrosis, and frailty (all nonatherosclerotic indications); the CABG trial (quercetin alone) tested ex vivo senescence and endothelial function, not atherosclerosis outcomes; no trial designed to test atherosclerotic cardiovascular event reduction with quercetin was identified; the meta‐analysis of 884 randomized micronutrient trials supported improvements in cardiovascular risk factors with quercetin, but these surrogate findings do not, by themselves, establish cardiovascular event reduction [50]	Low oral absorption (< 10%); rapid metabolism; nanocarrier and prodrug formulations in development	[20, 47–57]
Fisetin	Strawberries, apples (flavonol)	Senolytic (selective killing of confirmed senescent human endothelial cells with nonsenescent controls assayed in parallel); senomorphic action demonstrated only in nonvascular cells	Selective apoptosis of senescent cells, proapoptotic target not established (the BCL‐XL inhibition often attributed to fisetin was the mechanism of the separately tested compounds A1331852 and A1155463); p16INK4a pathway	None measured in confirmed senescent vascular cells; secreted IL‐6 and IL‐8 reduced in senescent nonvascular fibroblasts; adhesion molecules reduced in nonsenescent stressed endothelial cells	Reduced aortic sinus plaque and lipid area in high‐fat‐diet ApoE−/− mice with lower whole‐aorta p16, p21 and p53 but no senescent cell enumeration, and with the lesion effect confounded by concurrent lipid lowering [58]; lesion reduction with no senescence endpoint at all in three further models [59–61]; reduced aortic p16 and improved endothelial function in old non‐atherosclerotic mice, shown by ex vivo p16‐3MR clearance to be mediated by senolysis [62]; extended healthspan in aged mice [63]; senolysis inside an atherosclerotic plaque not demonstrated	No published human cardiovascular data; the chronic kidney disease trial is suspended and unpublished, the COVID‐19 trials were placebo‐controlled pilot studies of clinical recovery and safety, and the only published post‐COVID report is an uncontrolled cross‐sectional survey [64]; two directly vascular trials, in endothelial dysfunction and arterial stiffness and in peripheral artery disease, are registered but unreported	Low aqueous solubility; limited oral absorption	[58–69]
Resveratrol	Grapes, berries, red wine (stilbene)	Not established as senomorphic; senescence‐preventive and vascular protective (SASP output was not reduced by resveratrol alone in confirmed senescent human endothelial cells; reduced secreted IL‐8 is documented only in nonvascular fibroblasts)	SIRT1 activation; deacetylation of NF‐κB p65; PGC‐1*α*; FOXO	Not reduced by resveratrol alone in confirmed senescent human endothelial cells; secreted IL‐8 reduced in senescent nonvascular fibroblasts; adhesion molecules and ROS reduced in nonsenescent cells	Attenuated atherosclerotic lesion development in hyperlipidemic models, including a 52% reduction in aortic root lesion area and a 108% higher plaque collagen‐to‐macrophage ratio in APOE ∗ 3‐Leiden.CETP mice, with plasma cholesterol also 19% lower and no senescence endpoint measured [70]; improved endothelial function; reduced arterial stiffness; one report couples reduced VSMC senescence and SASP with reduced atherosclerosis in ApoE−/− mice [71]; prosenescent in primary human endothelial cells during chronic 10‐μM exposure [72]	Surrogate endpoints only: pooled flow‐mediated dilation +1.43% (95% CI 0.98–1.88) and lower ICAM‐1 across 21 arms from 17 randomized trials, with VCAM‐1, fibrinogen and PAI‐1 unchanged [73], but one randomized double‐blind trial found that resveratrol blunted exercise‐training benefits in aged men [74]; no plaque‐imaging, cardiovascular outcome or senescence endpoint; pharmacokinetics limit response in humans	Very low oral bioavailability (< 1% as parent compound); extensive glucuronidation and sulfation	[67, 70–78]
Curcumin	Curcuma longa rhizome (curcuminoid)	Not established as senomorphic; dose‐ and tissue‐dependent senotherapeutic (senolytic in nonvascular intervertebral disc cells; senescence‐inducing in human aortic vascular cells at cytostatic doses)	NF‐κB (IκBα stability, p65 phosphorylation); NLRP3 inflammasome; Nrf2; COX‐2	Not reduced by curcumin alone in confirmed senescent human endothelial cells; secreted SASP factors reduced in senescent nonvascular intervertebral disc cells	Reduced aortic sinus lesion area and plaque macrophage infiltration via TLR4 inhibition in high‐fat‐diet ApoE−/− mice [79], and reduced plaque area with improved lipid profile via lipocalin‐2 downregulation in Western diet‐fed ApoE−/− mice [80]; no senescence‐specific endpoint measured in any atherosclerosis experiment	Randomized trials with surrogate endpoints only: solid‐lipid curcumin improved endothelial function in healthy middle‐aged and older adults without changing arterial stiffness [81] and nano‐curcumin lowered hs‐CRP and lipoprotein(a) in type 2 diabetes with mild‐to‐moderate coronary artery disease [82]; curcuminoids reduced in‐hospital myocardial infarction after CABG [83], a peri‐procedural endpoint distinct from atherosclerosis and senescence; no senescence endpoint in any of these trials	< 1% systemic bioavailability of unmodified curcumin; piperine raised bioavailability approximately 20‐fold in human volunteers [84], and a lecithin phospholipid complex raised curcuminoid absorption approximately 29‐fold, although only phase‐2 metabolites were detectable in plasma [85]	[76, 79–93]

### 5.2. TMP: A Vascular Protective Agent Without Senescence‐Specific Evidence

TMP, also known as ligustrazine, is a bioactive alkaloid isolated from *Ligusticum chuanxiong* Hort. (Chuanxiong), a medicinal plant used for centuries in traditional Chinese medicine to treat cardiovascular and cerebrovascular disorders. TMP causes vasodilation, inhibits platelet aggregation, scavenges ROS, and dampens inflammatory signaling. Mechanistically, however, TMP does not meet the operational criteria for a senomorphic agent in vascular tissue. No published study has applied TMP to endothelial cells, VSMCs, or plaque macrophages that were first confirmed to be senescent, and no TMP atherosclerosis experiment has reported p16INK4a, p21CIP1, SA‐β‐gal, telomere‐associated foci, or a secreted SASP factor in the aortic wall; a PubMed search combining TMP or ligustrazine with aortic, vascular, endothelial, or smooth muscle terms and with senescence terms identified no study that examined senescence in the vessel wall. The one report in which TMP was given to cells characterized as senescent lies outside the vasculature: local delivery of TMP to the bone marrow of aging mice inhibited the senescent phenotype of LepR‐positive mesenchymal stem and progenitor cells through EZH2 and H3K27me3, an effect on the senescent state itself rather than selective silencing of secretion, and the accompanying anti‐inflammatory readout was reported for the bone marrow microenvironment rather than as secreted output from the isolated senescent cells [41]. TMP is therefore best described as a vascular protective, anti‐inflammatory, and antioxidant compound and a candidate for senescence‐specific testing, rather than as an established senomorphic agent [42].

TMP inhibits several inflammatory pathways that also drive the SASP, but every experiment establishing this was performed in cells that were not shown to be senescent, so these data support anti‐inflammatory rather than senomorphic activity. In brain microvascular endothelial cells injured by oxygen‐glucose deprivation and reoxygenation, TMP blocks NF‐κB activation, prevents nuclear translocation of the p65 subunit, and reduces transcription of IL‐6, IL‐8, and endothelial adhesion molecules. TMP also suppresses p38 MAPK phosphorylation, dampening the stress‐induced transcription of inflammatory mediators. Its antioxidant activity lowers ROS generation, which in turn limits oxidative DNA damage and activation of the DDR [43]. Because senescence was neither induced nor confirmed in any of these models, these observations establish general anti‐inflammatory, antioxidant, and endothelial protective activity rather than a senomorphic mechanism.

In animal models of atherosclerosis, TMP reduces plaque area, improves serum lipid profiles, and lowers circulating inflammatory markers: The relative aortic lesion area fell from 0.66 to 0.41 and 0.27 at 75 and 150 mg/kg in cholesterol‐fed rabbits [37], corrected plaque area was significantly reduced in ApoE−/− mice fed a high‐fat diet [38], and a systematic review of 12 studies comprising 258 animals confirmed a significant reduction in aortic lesion area together with lower total cholesterol, triglycerides, and LDL cholesterol [39]. None of these experiments measured a senescence‐specific endpoint, and the mechanisms catalogued in that systematic review are anti‐inflammatory, antioxidant, lipid‐modulating, endothelial protective, antiplatelet, antiproliferative, and antiangiogenic, with senescence and SASP modulation absent from the list [39]. At the cellular level, TMP protects endothelial cells from ox‐LDL‐induced dysfunction, preserves barrier integrity, and reduces adhesion molecule expression. A more recent finding shows that TMP also protects the endothelial glycocalyx, the carbohydrate‐rich coating that lines the vascular lumen, by inhibiting heparanase‐1 (HPSE1) expression downstream of the Toll‐like receptor 4 (TLR4)/NF‐κB axis. Because glycocalyx integrity controls monocyte adhesion and barrier function during early atherogenesis [40], this mechanism places TMP upstream of several stress signals capable of inducing senescence. Because the glycocalyx experiments used lipopolysaccharide‐stimulated rather than senescent endothelium [40], however, they support the evaluation of TMP as a candidate for senescence‐specific testing in atherosclerosis rather than its classification as a multi‐target senomorphic agent.

### 5.3. Quercetin: A Flavonoid With Senolytic Properties

Quercetin is a dietary flavonoid abundantly present in foods such as apples, onions, and berries. It demonstrates clear senolytic activity by targeting prosurvival signaling networks that are essential for senescent cell viability. Its mechanism involves interference with the PI3K–AKT pathway and a reduction in the expression of antiapoptotic BCL‐2 family proteins, thereby sensitizing senescent cells to apoptosis. The most widely studied senolytic regimen to date is the combination of dasatinib and quercetin (D + Q). This combination has shown efficacy in reducing senescent cell burden across multiple tissue types in preclinical models and in early‐phase human clinical trials [51, 52].

In the cardiovascular context, quercetin is one of the two compounds reviewed here—the other being fisetin—whose senolytic label is supported by direct evidence in vascular cells: At 10 μM, quercetin alone reduced the number of irradiation‐induced senescent human umbilical vein endothelial cells by approximately 50% over 3 days, while parallel cultures of nonsenescent cells increased two‐ to threefold in number, indicating selective killing of senescent cells [51]. Evidence for a separate senomorphic action of quercetin on vascular cells is much weaker. The reported reductions in SASP factors come from cells in which senescence was prevented rather than from cells first confirmed to be senescent, and inhibition of NF‐κB or activation of endogenous antioxidant defenses in stressed but nonsenescent cells does not by itself establish a senomorphic mechanism. Cellular studies in which quercetin was applied to human aortic endothelial cells (HAECs) and to macrophages exposed to ox‐LDL show that it protects these cells from acquiring senescence markers such as SA‐β‐gal activity and p16, p21, p53, and SERPINE1 expression. These are senescence prevention experiments rather than treatments of established senescent cells; no secreted SASP factor was reported in vitro, and apoptosis was reduced rather than increased, so they do not demonstrate suppression of SASP output from senescent cells [53]. Quercetin monotherapy nevertheless reduces plaque burden and improves endothelial function in atherosclerotic mice. Aortic lesion area was 46% smaller in apolipoprotein E–deficient mice given 50 μg/day for up to 6 weeks, an effect associated with reduced susceptibility of LDL to oxidation and aggregation and with higher serum paraoxonase activity [47], and 64 mg/kg/day attenuated lesion size in the aortic sinus and thoracic aorta while lowering aortic F2‐isoprostanes, vascular superoxide, and plasma soluble P‐selectin and augmenting vascular endothelial nitric oxide synthase activity [48]; neither of these studies measured a senescence endpoint, and quercetin also attenuated disease progression in high‐fat‐diet ApoE−/− mice in the study described above [53]. The clearest experiment pairing a lesion endpoint with an in vivo senescence‐specific endpoint under quercetin monotherapy reported an accumulation of senescent macrophages in plaque‐rich aortic tissue that quercetin reduced, together with suppression of key SASP factors and attenuated atherosclerosis through inhibition of p38 MAPK phosphorylation and p16 expression [49].

Translational support for quercetin’s potential benefits comes from recent clinical evidence. A randomized, double‐blind, placebo‐controlled trial in 97 patients (78 men) undergoing coronary artery bypass grafting found that preoperative quercetin supplementation reduced markers of vascular senescence and improved endothelium‐dependent vasodilation in internal thoracic artery tissue examined ex vivo [54]. These benefits were confined to male patients: in women, endothelium‐dependent relaxation did not improve and the circulating inflammatory protein profile shifted in a proinflammatory direction. Because the trial tested quercetin alone and assessed senescence as a tissue transcriptomic signature rather than as clearance of senescent cells, it demonstrates a sex‐dimorphic vascular effect of quercetin rather than senolysis in humans, and it argues for prespecifying sex as a stratification variable in the design of senotherapeutic trials. A significant challenge for quercetin’s clinical application is its limited bioavailability, characterized by rapid metabolism and modest absorption. This has motivated the development of advanced formulations, including liposomal encapsulations and prodrug derivatives, which may enhance its delivery and clinical efficacy [55]. Evidence for quercetin’s effects on cardiovascular risk factors should be distinguished from evidence of reduced atherosclerotic cardiovascular events. A systematic review and meta‐analysis of 884 randomized intervention trials evaluating 27 micronutrients among 883,627 participants identified moderate‐to‐high‐quality evidence for improvements in cardiovascular risk factors with quercetin. Across the micronutrients studied, clinical outcomes were heterogeneous, with benefits for some endpoints, neutral effects for others, and harmful effects associated with beta‐carotene supplementation [50]. Improvements in surrogate risk factors do not, by themselves, establish a reduction in cardiovascular events; the risk‐factor findings for quercetin should therefore not be interpreted as evidence of cardiovascular event reduction.

### 5.4. Fisetin: A Natural Senolytic Flavonol

Fisetin is a flavonol compound found in strawberries, apples, and other fruits. Preclinical studies have established its potent senolytic activity alongside a favorable safety profile. Fisetin treatment reduces senescent cell populations in various tissues and has been shown to extend healthspan in aged mice, alleviating age‐related pathologies like metabolic dysfunction and physical frailty. Mechanistically, fisetin selectively induces apoptosis in senescent but not proliferating human umbilical vein endothelial cells, while showing no senolytic activity against senescent IMR‐90 lung fibroblasts or primary human preadipocytes [65]. The proapoptotic target responsible has not been established; the BCL‐XL inhibition often attributed to fisetin was in fact the mechanism of the separately tested compounds A1331852 and A1155463 in that report [65]. The senolytic assignment has since been extended to arterial cells and causally linked to vascular benefit: in replicatively senescent human umbilical vein endothelial cells the half‐maximal inhibitory concentration of fisetin was 3.4 μM compared with 7.0 μM in nonsenescent control cells, and 1 μM fisetin reduced senescent cell viability with no significant effect on the viability of control cells; in replicatively senescent HAECs 1 μM fisetin lowered CDKN2A expression by 71%; and in 27‐month‐old mice intermittent oral fisetin lowered aortic p16 protein by 36%, with an ex vivo p16‐3MR genetic clearance experiment, demonstrating that the improvement in endothelium‐dependent dilation was mediated by senolysis rather than by an off‐target vasoactive effect [62]. Evidence for a senomorphic action is confined to nonvascular cells: Liposome‐encapsulated fisetin reduced secreted IL‐6 and IL‐8 from doxorubicin‐induced senescent WI‐38 lung fibroblasts without senolysis [66], and noncytotoxic fisetin lowered MMP‐9, MMP‐13, IL‐1β, and IL‐6 in senescent chondrogenic progenitor cells from osteoarthritic cartilage [67]; a comparable measurement in confirmed senescent vascular cells has not been reported [63].

When applied in models of atherosclerosis, fisetin treatment has been associated with reduced lesion development and improved vascular function, but only one such study also measured senescence. In ApoE−/− mice fed a high‐fat diet and given fisetin for 12 weeks, plaque area and lipid accumulation in the aortic sinus were significantly reduced and aortic p16, p21, and p53 were downregulated; the senescence readout was whole‐aorta expression without senescent cell enumeration or cell‐type attribution, and the same treatment also downregulated PCSK9 and LOX‐1, with atorvastatin as the positive comparator, so the lesion effect cannot be separated from an effect on lipid handling [58]. Three further studies reported reduced lesions with no senescence endpoint at all, attributing the effect to FXR‐mediated hepatic cholesterol metabolism and transintestinal cholesterol excretion [59], to inhibition of ferroptosis‐related oxidative stress [60], and, in streptozotocin‐diabetic LDLR−/− mice, to inhibition of the CD36/fibrosis pathway in a study whose primary focus was renal injury [61]; each of these also reported lower serum lipids. The reductions in adhesion molecules and inflammatory cytokines attributed to fisetin in vascular cells derive from high‐glucose‐ and ox‐LDL‐stressed human umbilical vein endothelial cells in which senescence was never assessed, and therefore represent general endothelial protection rather than senomorphic activity [68, 69]. Senolysis within an atherosclerotic plaque has not been demonstrated for fisetin. Fisetin is therefore best classified as a senolytic with additional general anti‐inflammatory activity; its senomorphic credentials rest on nonvascular cells, and no study has yet demonstrated clearance of senescent cells from within an atherosclerotic plaque. The translational potential of fisetin is being explored in registered trials in age‐related conditions, but none has yet reported a cardiovascular endpoint. The chronic kidney disease trial is listed as suspended and remains unpublished; the COVID‐19 trials were placebo‐controlled pilot studies of clinical recovery and safety; and the only published human report in the post‐COVID setting is an uncontrolled cross‐sectional survey of a long‐COVID clinic in which 28 of 44 respondents who had taken fisetin rated it helpful, with no senescence and no cardiovascular measure [64]. Two directly vascular trials, one in endothelial dysfunction and arterial stiffness in older adults and one in mobility impairment in peripheral artery disease, are registered but as yet unreported, so no human cardiovascular efficacy data for fisetin have been published.

### 5.5. Resveratrol: A SIRT1‐Activating Vascular Protective Agent With Unconfirmed Senomorphic Activity

Resveratrol is a stilbenoid polyphenol present in grapes, berries, and red wine. Its cardiovascular protective effects are largely attributed to the activation of SIRT1, a NAD + ‐dependent deacetylase, and the subsequent modulation of downstream pathways involving NF‐κB, oxidative stress, and mitochondrial function. SIRT1 deacetylates and inactivates the p65 subunit of NF‐κB, leading to reduced transcription of inflammatory genes. Additionally, SIRT1 activation enhances mitochondrial biogenesis via PGC‐1α and boosts cellular antioxidant defenses through the activation of FOXO transcription factors [75].

This mechanism is often taken to classify resveratrol as a senomorphic agent, but the vascular evidence does not support that assignment. In the only study to test resveratrol against the operational criterion in confirmed senescent human endothelial cells, using replicatively senescent and doxorubicin‐induced senescent HUVECs with more than 60% SA‐β‐gal positivity and elevated p16INK4a, and treating at concentrations chosen to preserve viability, resveratrol alone did not reduce IL‐1β or IL‐6 in both senescence models; only a three‐compound combination did, and its effect on secreted IL‐1β and IL‐6 was confined to the replicatively senescent cells, although the exposure lasted only three hours [76]. Reduction of SASP output by resveratrol alone has been shown outside the vasculature, in MRC5 lung fibroblasts, where chronic treatment lowered IL‐8 in conditioned medium and blunted the proangiogenic activity of that medium [77], and in osteoarthritic chondrogenic progenitor cells, in which the senescence index also fell [67]. Chronic exposure to 10 μM resveratrol has moreover been reported to induce, rather than prevent, senescence in primary human endothelial cells through NADPH Oxidases 1 and 4 [72]. Resveratrol is therefore better described as a vascular protective and largely senescence‐preventive compound whose senomorphic activity in the vessel wall is unconfirmed; a single recent report that it attenuates smooth muscle cell senescence and the SASP through the GATA4/sortilin axis in ApoE−/− mice is the only supportive vascular evidence, and because senescence itself was attenuated, it does not separate SASP suppression from prevention of senescence [71]. In endothelial cell models, resveratrol treatment mitigates oxidative stress‐induced senescence, helps preserve nitric oxide production, and reduces the expression of adhesion molecules and inflammatory cytokines. In VSMCs, it inhibits pathological phenotypic switching and lowers MMP expression. Animal studies show that resveratrol supplementation improves endothelial function, reduces vascular stiffness, and decreases atherosclerotic lesion burden; in APOE∗3‐Leiden.CETP mice, for example, resveratrol reduced aortic root lesion area by 52% and raised the plaque collagen‐to‐macrophage ratio by 108%, although plasma cholesterol also fell by 19% and no senescence‐specific endpoint was measured, which is typical of the resveratrol atherosclerosis literature [70, 78].

Despite its promising preclinical profile, the clinical translation of resveratrol is hampered by pharmacokinetic challenges, notably low oral bioavailability and rapid metabolism. The biologically active trans‐resveratrol isomer undergoes extensive first‐pass metabolism via glucuronidation and sulfation, resulting in low plasma concentrations of the parent compound. Strategies to overcome these limitations are under active investigation; these include the development of novel delivery systems, the synthesis of structural analogs with improved pharmacokinetics, and the use of combination regimens [78]. Human cardiovascular data remain confined to surrogate endpoints and are internally inconsistent: A meta‐analysis of 21 arms from 17 randomized controlled trials found flow‐mediated dilation improved by 1.43% (95% CI 0.98–1.88) and ICAM‐1 lowered, with VCAM‐1, fibrinogen, and PAI‐1 unchanged [73], whereas a randomized, double‐blind trial in aged men found that resveratrol blunted rather than enhanced the gains in maximal oxygen uptake, mean arterial pressure, and lipid profile normally produced by exercise training [74]. No trial has assessed plaque imaging, cardiovascular events, or any senescence endpoint.

### 5.6. Curcumin: An NF‐κB Inhibitor Without Established Senomorphic Activity in Vascular Cells

Curcumin, the primary polyphenolic constituent of turmeric (*Curcuma longa*), possesses a broad spectrum of biological activities, including anti‐inflammatory, antioxidant, and metabolic‐modulating effects. It is a potent inhibitor of NF‐κB activation, employing multiple mechanisms such as preventing IκBα degradation, reducing p65 nuclear translocation, and decreasing p65 phosphorylation. In addition to NF‐κB, curcumin also suppresses the activation of the NLRP3 inflammasome, inhibits the expression of cyclooxygenase‐2 (COX‐2), and activates the Nrf2 antioxidant pathway. These concerted actions work to reduce the inflammatory signaling and oxidative stress that are fundamental drivers of the SASP [86].

The vascular senescence data for curcumin are preventive and inconsistent rather than senomorphic. Pretreatment of human umbilical vein endothelial cells before a hydrogen peroxide challenge lowered the SA‐β‐gal‐positive fraction and p21 through SIRT1, but curcumin was given before the senescence stimulus and no secreted SASP factor was measured [87]. In cells that were already senescing, 0.1 and 1 μM curcumin neither postponed replicative senescence nor protected against doxorubicin‐induced senescence in primary human VSMCs and slightly accelerated endothelial replicative senescence [88]. When a standardized turmeric extract was applied as a single agent to human endothelial cells first confirmed to be senescent, it did not reduce IL‐1β and IL‐6 in both senescence models, and only the three‐compound combination was effective [76]. The findings of reduced p16INK4a‐positive cells and lower secreted IL‐6 derive from human intervertebral disc cells, in which curcumin acted as a senolytic, selectively inducing apoptosis in p16INK4a‐positive, Ki‐67‐negative cells and raising apoptosis in cells from degenerate but not from nonmildly degenerate discs, and not from vascular cells [89]. The senolytic activity otherwise described in this literature belongs to synthetic curcumin analogs such as EF24, which was itself not selective in irradiation‐induced senescent endothelial cells, rather than to curcumin [90]. An important caveat is the observation of dose‐dependent effects: At higher, cytostatic concentrations, curcumin induces senescence in both primary human VSMCs and endothelial cells derived from the aorta, through a DNA damage‐ and ATM‐independent mechanism, and the authors of that work explicitly cautioned that increasing curcumin bioavailability should be approached carefully because senescence induction is a possible side effect [91]. This highlights the critical importance of dose optimization. In vivo, curcumin supplementation in atherosclerotic animal models reduces lesion area and inflammatory cell infiltration and improves the lipid profile through inhibition of TLR4 signaling in high‐fat‐diet ApoE−/− mice [79] and downregulation of lipocalin‐2 in Western diet‐fed ApoE−/− mice [80]. No senescence endpoint was measured in either experiment, so the lesion effect is attributable to anti‐inflammatory and lipid‐lowering actions and cannot be assigned to senotherapy [92]. The available in vivo evidence that dietary curcumin lowers senescence markers comes from the liver of aged mice rather than from the vessel wall [93].

Curcumin, like resveratrol, has limited therapeutic effects due to unfavorable pharmacokinetic characteristics such as low absorption, fast metabolism, and widespread elimination. As a solution to these issues, various improved formulations have been developed. These include phospholipid complexes, delivery systems in the form of nanoparticles, structural analogs, and combination products containing bioavailability enhancers such as piperine, which raised curcumin bioavailability by approximately 20‐fold in human volunteers given 2‐g curcumin with 20‐mg piperine [84], and lecithin‐based phospholipid complexes, which increased total curcuminoid absorption approximately 29‐fold, although only Phase‐2 metabolites were detectable in plasma and concentrations remained below those required for the inhibition of most anti‐inflammatory targets of curcumin [85]. One direct test supports this idea only in part: In human endothelial cells first confirmed to be senescent, a combination of bioavailable curcumin, resveratrol, and β‐caryophyllene reduced IL‐1β and IL‐6 expression in both replicative and doxorubicin‐induced senescence models, whereas none of the three compounds achieved this alone; the reduction in secreted IL‐1β and IL‐6 was, however, confined to the replicative model [76].

Table 2 stratifies this evidence by experimental model, senescence‐specific endpoint, atherosclerosis endpoint, and human cardiovascular data. None of the five compounds has been tested against a plaque‐imaging or cardiovascular event endpoint in atherosclerotic disease, and no human study identified here measured a senescence endpoint except the quercetin bypass surgery trial. Human data are otherwise limited to surrogate vascular function and circulating biomarkers for quercetin, resveratrol, and curcumin and are absent for fisetin and TMP, neither of which has a reported trial with a vascular endpoint. The preclinical gaps are equally uneven: No senescence‐specific endpoint has ever been measured for TMP in a vascular cell or an atherosclerotic vessel, or for curcumin in any atherosclerosis model. Mechanistic plausibility therefore remains well ahead of demonstrated clinical benefit.

**TABLE 2:** Stratification of the evidence for each natural product senotherapeutic by experimental model, senescence‐specific endpoint, atherosclerosis endpoint, and human cardiovascular data.

Compound	Experimental model	Senescence‐specific endpoint	Atherosclerosis endpoint	Human cardiovascular evidence	Level of evidence
Tetramethylpyrazine (TMP, ligustrazine)	In vitro: ox‐LDL‐stimulated rat aortic endothelial cells [37], oxygen‐glucose‐deprived mouse brain microvascular endothelial cells [43], and lipopolysaccharide‐stimulated endothelial cells [40]; senescence was neither induced nor confirmed in any of these. Confirmed senescent cells have been studied only outside the vasculature, in LepR‐positive bone marrow mesenchymal stem/progenitor cells from 20‐month‐old mice [41]. In vivo: cholesterol‐fed rabbits [37] and high‐fat‐diet ApoE−/− mice [38], pooled in a meta‐analysis of 12 studies and 258 animals [39].	Not measured in any vascular cell or atherosclerotic vessel. A PubMed search combining tetramethylpyrazine or ligustrazine with aorta, atherosclerosis, endothelial, or smooth muscle and with senescence, p16, p21, SA‐β‐gal, or SASP identified no study that assessed senescence: no SA‐β‐gal, p16INK4a, p21CIP1, γ‐H2AX, or SASP secretome data exist for TMP in endothelial cells, vascular smooth muscle cells, plaque macrophages, or aortic tissue. SA‐β‐gal‐positive and p16INK4a‐positive fractions fell, p16 and p21 transcripts fell, and BrdU incorporation rose only in confirmed senescent bone marrow mesenchymal stem/progenitor cells, where the secretory readout was whole‐marrow TNF‐α, IL‐1β, and IL‐6 content rather than secretion by the senescent cells themselves, and where proliferation was restored rather than the cells killed [41]. The direction of effect also reverses by cell type: In hepatic stellate cells, TMP induces senescence [46].	Measured and positive. In cholesterol‐fed rabbits the relative aortic lesion area ratio fell from 0.66 ± 0.058 in untreated animals to 0.41 ± 0.042 and 0.27 ± 0.047 at 75 and 150 mg/kg, with a lower intima/media thickness ratio and fewer intimal monocytes [37]; corrected plaque area was reduced in high‐fat‐diet ApoE−/− mice [38]; and meta‐analysis of 12 studies and 258 animals confirmed reduced aortic lesion area with favorable lipid effects [39]. None reported plaque‐composition, fibrous‐cap, necrotic‐core, or calcification data, and none measured a senescence endpoint.	No atherosclerosis‐relevant human evidence. We identified no trial of TMP with an atherosclerosis, cardiovascular outcome, or senescence endpoint, and none testing TMP as monotherapy for atherosclerosis. Ligustrazine reaches patients mainly in compound preparations assessed on symptom and biomarker scales: A network meta‐analysis of seven blood‐activating Chinese medicine injections in acute ischemic stroke (87 randomized trials, 8580 patients) evaluated the ligustrazine‐containing injection only on NIHSS score, Barthel index, platelet aggregation, fibrinogen and high‐sensitivity C‐reactive protein [94], and a 30‐patient controlled trial in chronic stable angina tested a Danlou tablet plus Salvia miltiorrhiza–ligustrazine injection combination on lipids, cytokines, and gut microbial metabolites [95].	L3: animal atherosclerosis model with a lesion endpoint, senescence‐specific endpoint not measured. L4, L5a, and L5b are not reached. L2 is reached only outside the cardiovascular system, and every vascular in vitro study sits at L1 because the cells were never shown to be senescent. On the three‐tier test, TMP is therefore neither senolytic nor senomorphic in vascular tissue; the demonstrated actions are those of a vascular protective, anti‐inflammatory, and antioxidant agent.
Quercetin	In vitro: radiation‐induced senescent human umbilical vein endothelial cells, with proliferating nonsenescent cultures assayed in parallel, and senescent bone marrow mesenchymal stem cells from progeroid mice, quercetin alone [51]; ox‐LDL‐exposed human aortic endothelial cells and RAW264.7 macrophages, quercetin applied against the senescence stimulus rather than to established senescent cells [53]. In vivo: quercetin monotherapy in ApoE−/− mice [47–49, 53] and dasatinib plus quercetin in Western diet‐fed ApoE−/− mice [20]. Human: randomized, double‐blind trial in 97 coronary artery bypass patients, 78 of them men [54].	Measured at every preclinical level. Quercetin alone was selectively cytotoxic to confirmed senescent human endothelial cells: At 10 μM, nonsenescent HUVEC cultures increased two‐ to threefold in cell number between Days 0 and 3, while parallel senescent cultures fell by 50%, and senescent bone marrow mesenchymal stem cells were likewise depleted [51]. In ApoE−/− mice, quercetin monotherapy reduced the burden of senescent macrophages in plaque‐rich aortic tissue and lowered key SASP factors through p38 MAPK/p16; because ox‐LDL‐induced senescence was itself reversed in parallel macrophage cultures, this reflects suppression of the senescent phenotype rather than demonstrated selective killing within plaque [49]. Consistent with this, quercetin increased rather than decreased the viability of ox‐LDL‐exposed human aortic endothelial cells [53]. With dasatinib plus quercetin, telomere‐associated foci‐positive cells were cleared from the aortic media but not from within intimal plaque [20]. In humans, only an internal thoracic artery transcriptomic senescence signature was assessed, not senescent cell clearance [54].	Measured and positive for quercetin monotherapy: aortic lesion area reduced by 46% [47]; lesion size reduced in the aortic sinus and thoracic aorta at 64 mg/kg/day [48]; atherosclerosis attenuated alongside depletion of plaque senescent macrophages [49]; progression attenuated in high‐fat‐diet ApoE−/− mice [53]. With dasatinib plus quercetin, by contrast, intimal plaque size, plaque lipid content, F4/80 macrophage burden, and plaque fibrosis were all unchanged, the only structural benefit being a large reduction in intimal plaque calcification and Osterix signaling [20].	Primary endpoints were surrogate and ex vivo. In 97 bypass patients, quercetin 500 mg twice daily improved acetylcholine‐induced relaxation of internal thoracic artery segments overall (*p* = 0.049) and in men (*p* = 0.043) but not in women (*p* = 0.852), in whom the circulating inflammatory protein profile shifted in a proinflammatory direction; C‐reactive protein showed only a trend (*p* = 0.073) [54]. New‐onset postoperative atrial fibrillation was lower with quercetin (4% versus 18%, *p* = 0.033), but the prespecified primary outcomes were ex vivo endothelial function and inflammation, so this is a secondary observation. We identified no trial designed to test plaque progression or atherosclerotic cardiovascular event reduction with quercetin; the meta‐analysis identified moderate‐ to high‐quality evidence for improvements in cardiovascular risk factors, but these surrogate findings do not, by themselves, establish a reduction in cardiovascular events [50].	L5a: human surrogate and ex vivo endpoint, with L2 and L4 also genuinely met and L5b absent. This is the only compound in the review that satisfies the strict senolytic definition as a single agent in human endothelial cells, and the only one reaching L5a. Senomorphic action in vascular cells is not established: No study has shown reduced SASP output from confirmed senescent vascular cells that were not simultaneously killed or prevented from becoming senescent, and selective killing of senescent cells inside an atherosclerotic plaque has never been demonstrated.
Fisetin	In vitro: replicatively senescent human umbilical vein and aortic endothelial cells with nonsenescent controls assayed in parallel [62] and radiation‐induced senescent HUVECs [65]; doxorubicin‐induced senescent WI‐38 lung fibroblasts and A549 lung carcinoma cells treated with liposomal fisetin [66]; high‐glucose‐stressed HUVECs never shown to be senescent [68]. In vivo atherosclerosis: high‐fat‐diet ApoE−/− mice [58] and streptozotocin‐diabetic LDLR−/− mice [61]. In vivo aging vasculature without lesions: 27‐month‐old wild‐type and p16‐3MR mice [62]. Human: one randomized adjunct trial in acute ischemic stroke [96], plus two registered vascular trials that remain unreported.	Measured, and the strongest of the five in vitro, although the selectivity is modest and cell type‐dependent. Fisetin selectively induced apoptosis in senescent but not proliferating human umbilical vein endothelial cells, while not being senolytic in senescent IMR90 lung fibroblasts or primary human preadipocytes [65]. Its half‐maximal inhibitory concentration was 3.4 ± 0.3 μM in senescent versus 7.0 ± 0.4 μM in nonsenescent HUVECs, and at 1 μM, viability fell to 91 ± 1% in senescent cultures while nonsenescent controls were unaffected at 97 ± 1% (*p* = 0.003); in human aortic endothelial cells, the same study measured transcripts only, lowering CDKN2A by 71% with no effect on CDKN1A [62]. In aged nonatherosclerotic mice, aortic p16INK4a protein fell by 36%, and ex vivo p16‐3MR genetic clearance of senescent cells produced no further improvement in endothelium‐dependent dilation of carotid arteries from fisetin‐treated animals, although it did in vehicle‐treated animals, which is causal evidence that the functional benefit was mediated by reduced senescence; aortic SASP transcripts were only partly reduced, with both proteinases and one of two chemokines reaching significance, while the cytokine and growth factor transcripts did not. In the atherosclerosis model, only whole‐aorta p16, p21, and p53 proteins were measured by Western blot [58], with no SA‐β‐gal, no senescent cell enumeration, no cell‐type attribution, and no SASP; senescence was not measured at all in the other three in vivo atherosclerosis studies. SASP output has never been measured in confirmed senescent vascular cells; the only senomorphic data come from lung fibroblasts and a lung carcinoma line, where liposomal fisetin reduced secreted IL‐6 and IL‐8 but showed no senolytic effect [66].	Measured. Aortic sinus plaque area and lipid content fell and plaque collagen rose, but each of these effects, and the reductions in p16, p21, and p53, was statistically indistinguishable from atorvastatin (*p* > 0.05), and fisetin also lowered total, LDL and VLDL cholesterol and oxidized LDL, so the lesion effect is confounded by lipid lowering [58]; aortic root lipid deposition was reduced through hepatic and transintestinal cholesterol handling [59]; lesion formation was reduced through inhibition of ferroptosis‐related oxidative stress, again with serum lipid lowering [60]; diabetes‐exacerbated atherosclerosis was attenuated [61]. No necrotic‐core, fibrous‐cap, macrophage‐burden, calcification, or intraplaque senescent cell data exist for fisetin.	No atherosclerosis‐relevant human evidence. The only published randomized trial with a clinical cardiovascular endpoint is a single‐center study in acute ischemic stroke, in which fisetin added to recombinant tissue plasminogen activator lowered National Institutes of Health Stroke Scale scores and serum MMP‐2, MMP‐9, and C‐reactive protein [96]; it measured no vascular, atherosclerosis, or senescence endpoint. The two directly vascular trials are registered but unreported: NCT06133634 (endothelial function and arterial stiffness in older adults, Phase 1/2, *n* = 70, active, not recruiting) and NCT06399809 (FIRST, peripheral artery disease, Phase 2, *n* = 34, recruiting, primary endpoint 6‐min walk distance). The chronic kidney disease trial (NCT03325322) is suspended and unpublished; of the 36 fisetin studies registered on ClinicalTrials.gov, none is registered for post‐COVID‐19 frailty, and none of the COVID‐19 trials measures a cardiovascular endpoint.	L4, but qualified: The single study reaching it pairs a lesion endpoint with whole‐tissue cyclin‐dependent kinase inhibitor protein only, and its lesion effect is confounded by concurrent lipid lowering and is no greater than atorvastatin’s. Independently, fisetin holds a strong L2 in human endothelial cells. L5a and L5b are absent for atherosclerosis, and the one clinical outcome trial lies outside atherosclerotic plaque biology. The senolytic designation is justified for endothelial cells; senomorphic action in vascular cells is not established, because SASP output has only ever been measured in nonvascular cells, where fisetin proved senomorphic but not senolytic.
Resveratrol	In vitro: human umbilical vein endothelial cells in replicative and doxorubicin‐induced senescence, confirmed by SA‐β‐gal positivity above 60% and elevated p16INK4a, treated for 3 h at viability‐preserving concentrations [76]; primary human endothelial cells under chronic 10‐μM exposure [72]; vascular smooth muscle cells with GATA4‐driven senescence [71]; senescent human MRC5 lung fibroblasts after 5 weeks of 5‐μM exposure [77]. In vivo: high‐fat‐diet ApoE−/− mice with adeno‐associated virus‐mediated GATA4 overexpression [71] and cholesterol‐fed APOE ∗ 3‐Leiden.CETP mice [70]. Human: small randomized trials with vascular function endpoints.	Measured, and unsupportive in vascular cells. In HUVECs first confirmed to be senescent, no single compound, resveratrol included, reproduced across both senescence models the reduction in IL‐1β and IL‐6 achieved by a three‐compound mixture, and the mixture itself lowered secreted IL‐1β and IL‐6 only in replicative and not in doxorubicin‐induced senescence; exposure lasted 3 h, so this is an absence of demonstrated effect rather than a firmly excluded one [76]. One unreplicated report describes reduced smooth muscle cell senescence and SASP through GATA4/sortilin, but senescence itself was attenuated, so SASP suppression cannot be separated from prevention of senescence entry [71]. Reduction of SASP output by resveratrol alone, measured as secreted IL‐8 in conditioned medium, is documented only in nonvascular fibroblasts [77]. Counterdirectionally, chronic 10 μM resveratrol was itself prosenescent in primary human endothelial cells through NADPH Oxidases 1 and 4 [68]. Selective killing of senescent cells has not been reported.	Measured and consistently positive, but almost always without a senescence endpoint: Aortic root lesion area was reduced by approximately 52%, and the plaque collagen‐to‐macrophage ratio rose by approximately 108%, with senescence not assessed [70]. A lesion endpoint and a senescence‐specific endpoint appear together only in the single 2026 study, and there is a GATA4‐overexpression ApoE−/− model rather than in conventional diet‐induced disease [71].	Surrogate endpoints only, with no senescence endpoint and an internally discordant signal. Across 21 arms from 17 randomized trials, flow‐mediated dilation improved by 1.43% (95% CI 0.98–1.88) and ICAM‐1 fell, whereas VCAM‐1, fibrinogen, and plasminogen activator inhibitor‐1 were unchanged [73]. In a randomized, placebo‐controlled trial in 27 aged men, 250 mg/day resveratrol blunted rather than enhanced exercise‐training gains in maximal oxygen uptake, mean arterial pressure, and blood lipids [74]. We identified no plaque‐imaging or cardiovascular outcome trial.	L4, but only through a single unreplicated 2026 study; the replicated animal base is L3. The intermediate rung is missing because L2 is not met in vascular cells and is met only in nonvascular fibroblasts. L5a rests on surrogate endothelial function, with one trial reporting harm, and L5b is absent. The evidence supports a senescence‐preventive and vascular protective agent rather than an established senomorphic one, and reported suppression of SASP secretion by resveratrol in senescent cells is not upheld by the only study that has tested it directly in confirmed senescent vascular cells.
Curcumin	In vitro: primary human aortic vascular smooth muscle and endothelial cells at cytostatic doses [91] and at 0.1 and 1 μM during replicative and doxorubicin‐induced senescence [88]; HUVECs pretreated before a 100 μM H_2_O_2_ challenge [87]; HUVECs first confirmed to be senescent, treated for 3 h with a bioavailable curcumin extract [76]; human nucleus pulposus and annulus fibrosus cells from degenerate and minimally degenerate discs [89]. In vivo: high‐fat‐diet ApoE−/− mice given 0.1% w/w curcumin for 16 weeks [79] and Western diet‐fed ApoE−/− mice given 40–80 mg/kg/day for 12 weeks [80]. Human: randomized trials with vascular function, biomarker, and perioperative endpoints.	Measured in vascular cells, and unsupportive. Pretreatment before an H_2_O_2_ challenge lowered the SA‐β‐gal‐positive fraction and p21 through SIRT1, with no secreted SASP factor assayed and no effect of curcumin alone on unstressed cells, so this is prevention rather than senomorphic action [87]. In cells already senescing, 0.1 and 1 μM curcumin neither postponed replicative senescence nor protected against doxorubicin‐induced senescence in human aortic vascular smooth muscle cells, and slightly accelerated endothelial replicative senescence [88], while cytostatic doses induced senescence in both vascular cell types [91]. In HUVECs first confirmed to be senescent, single‐agent curcumin did not reproduce across both senescence models the IL‐1β and IL‐6 reduction achieved by a three‐compound mixture [76]. Selective apoptosis of p16INK4a‐positive, Ki‐67‐negative cells, with caspase‐3/7 activity and apoptosis rising in cells from degenerate but not from minimally degenerate tissue and with secreted IL‐6 reduced approximately threefold, is documented only in intervertebral disc cells; there, secreted IL‐8, MMP‐3, and MMP‐13 fell only nonsignificantly, the larger reductions being transcript‐level [89]. Not measured in any atherosclerosis model or human study.	Measured. Reduced aortic sinus lesion extent and plaque macrophage infiltration, with lower aortic IL‐1β, TNF‐α, VCAM‐1, ICAM‐1, and NF‐κB activity [79], and reduced plaque area at 60 and 80 but not 40 mg/kg/day, with an improved serum lipid and inflammatory profile [80]. No senescence‐specific endpoint was measured in either experiment, so the lesion effect is attributable to anti‐inflammatory and lipid‐lowering actions and cannot be assigned to senotherapy.	Surrogate vascular endpoints plus one nonatherosclerotic clinical outcome. Solid‐lipid curcumin 2000 mg/day for 12 weeks in 39 healthy middle‐aged and older adults increased forearm blood flow to acetylcholine by 37% and brachial flow‐mediated dilation by 36%, with no change in aortic pulse wave velocity, carotid compliance, or circulating oxidative and inflammatory biomarkers [81]; nanocurcumin 80 mg/day for 90 days in 64 patients with Type 2 diabetes and mild‐to‐moderate coronary artery disease lowered high‐sensitivity C‐reactive protein and lipoprotein(a) [82]. In 121 patients undergoing coronary artery bypass grafting, curcuminoids 4 g/day reduced in‐hospital myocardial infarction from 30.0% to 13.1% (adjusted hazard ratio 0.35, 95% CI 0.13–0.95, *p* = 0.038), a periprocedural ischemia–reperfusion endpoint rather than an atherosclerosis endpoint [83]. We identified no plaque‐imaging trial, and no human study measured a senescence endpoint.	L5a: human surrogate vascular endpoints are met, but L4 is entirely absent and the atherosclerosis evidence stops at L3. L5b is not reached for atherosclerosis, because the one hard‐outcome trial concerns periprocedural infarction after bypass surgery, which is mechanistically unrelated to senescence. L2 is met only in intervertebral disc cells, where curcumin behaved as a senolytic, whereas the corresponding vascular tests are negative or counterdirectional for curcumin alone. Since no senescence‐specific level is attained in vascular tissue, senomorphic action in vascular cells is not established, and curcumin is better described as a dose‐ and tissue‐dependent senotherapeutic whose demonstrated vascular benefit is anti‐inflammatory, antioxidant, and nitric oxide–mediated.

*Note:* Because no standard evidence hierarchy exists for preclinical senescence research, levels are defined for this review as follows: L1, in vitro only, in cells not confirmed to be senescent; L2, in vitro in experimentally confirmed senescent cells, with a senescence‐specific endpoint measured; L3, animal atherosclerosis model with a lesion endpoint but no senescence endpoint; L4, animal atherosclerosis model with both a lesion endpoint and a senescence‐specific endpoint; L5a, human evidence with a surrogate or ex vivo vascular endpoint; L5b, human clinical cardiovascular outcome data in atherosclerotic disease. Each compound is assigned the highest level it genuinely reaches, and levels that are skipped or absent are stated explicitly rather than inferred. Clinical data obtained in cardiovascular indications that are not driven by atherosclerotic plaque, such as periprocedural myocardial injury or acute ischemic stroke, are recorded but are not counted toward L5b. Studies are identified by first author, year, and PubMed identifier where they are not already numbered in the reference list.

Whether any of these compounds reach the vessel wall is a separate question from whether they are active in culture, and four formulation strategies dominate the attempts to answer it. Absorption enhancers and phospholipid complexes, described above for curcumin, raise systemic exposure roughly 20‐ to 29‐fold, yet only Phase‐2 metabolites became measurable and plasma concentrations stayed below those needed to engage most curcumin targets [84, 85]. Liposomes have delivered fisetin to senescent fibroblasts [66], and nanotheranostic and microneedle platforms are in preclinical development for quercetin [55]. Prodrug design has gone furthest as a senescence‐directed strategy: An orally absorbed, self‐assembling prodrug activated by the β‐galactosidase overexpressed in senescent cells selectively killed senescent retinal pigment epithelium in mice, though not in vascular tissue [97]. Carriers can also reach plaque. Simvastatin‐loaded high‐density lipoprotein, polymeric micelle, and liposomal formulations accumulated in plaques of Apolipoprotein E–deficient mice on quantitative biodistribution [98], and macrophage membrane‐coated nanoparticles carrying resveratrol, alone or with pitavastatin, reached lesions and reduced plaque area [99, 100]. These are carrier‐directed studies. For conventional oral dosing, we identified no report of the concentration achieved by any of the five compounds in arterial wall or plaque tissue, and none relating a measured tissue concentration to a senescence endpoint. Higher plasma exposure therefore remains a proxy for, not a demonstration of, delivery to the target site.

## 6. Preclinical Evidence for Anti‐SASP Interventions in Atherosclerosis

### 6.1. In Vitro Models and Readouts

Controlled cell culture systems offer a fundamental platform for elucidating the mechanisms underlying cellular senescence and for the preliminary screening of potential senotherapeutic agents. Primary human vascular cells, such as HAECs, coronary artery endothelial cells (HCAECs), and aortic smooth muscle cells (HASMCs), can be induced into senescence through various established methods. These include replicative exhaustion through serial passaging, exposure to oxidative stress (e.g., hydrogen peroxide, H_2_O_2_), stimulation with inflammatory cytokines (e.g., TNF‐α, IL‐1β), or treatment with atherogenic stimuli like ox‐LDL. The resultant senescent state is confirmed using a panel of standard assays. These typically encompass the histochemical detection of SA‐β‐gal activity, immunofluorescence or immunohistochemical staining for established senescence effectors like p16^INK4a^ and p21^CIP1^, quantification of γ‐H2AX foci as a marker of persistent DNA damage, and flow cytometric analysis to confirm cell cycle arrest [5, 53].

Characterization of the accompanying SASP is a critical component of these in vitro evaluations. This is commonly achieved through multiplexed immunoassays (e.g., ELISA, Luminex) that quantify the levels of secreted cytokines, chemokines, and growth factors present in the conditioned media of senescent cells. To gain a broader view of the transcriptional reprogramming, gene expression profiling via reverse transcription quantitative polymerase chain reaction (RT‐qPCR) or RNA sequencing is employed. Furthermore, functional assays are utilized to assess the downstream biological consequences of the SASP. These assays evaluate processes such as the enhanced adhesion of monocytes to monolayers of senescent endothelial cells, the accelerated formation of macrophage‐derived foam cells, changes in VSMC migration and proliferation, and alterations in endothelial tube formation capacity as a proxy for angiogenic potential. Together, this multifaceted experimental approach allows for a comprehensive assessment of how natural compounds influence both the initiation of the senescent state and the subsequent secretory phenotype [12]. Figure 3 shows schematically the downstream effects of SIRT1 activation on NF‐κB p65 signaling, SASP vesicle secretion, and mitochondrial oxidative stress, the pathway through which resveratrol is proposed to act.

**FIGURE 3:**
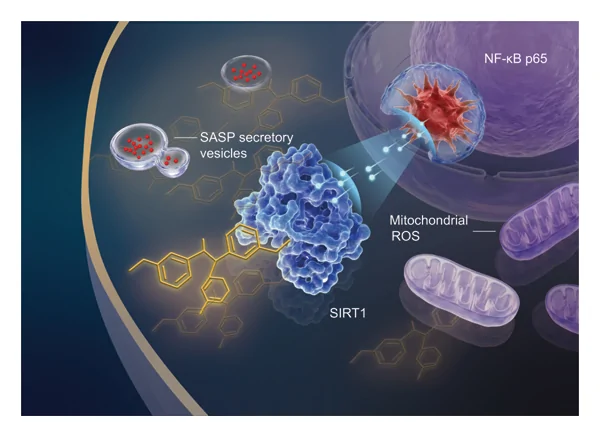
SIRT1‐dependent restraint of NF‐κB p65 signaling, mitochondrial ROS production, and SASP secretory vesicle release in a vascular cell.

Schematic of the intracellular circuit through which SIRT1 restrains the senescence‐associated secretory program in a vascular cell. SIRT1 deacetylates and inactivates the p65 subunit of NF‐κB, lowering the transcription of the inflammatory genes that encode SASP factors. SIRT1 activity is also associated with reduced mitochondrial ROS output, removing an oxidative stimulus that would otherwise sustain NF‐κB p65 activation and reinforce the secretory program. The combined effect is a reduction in the loading and release of SASP secretory vesicles, which carry cytokines, chemokines, and matrix‐degrading enzymes into the extracellular space. Arrows indicate the direction of regulation. The diagram is a conceptual summary and is not drawn to scale; mechanistic detail is presented in Sections 3.2 and 5.5. Abbreviations: NF‐κB, nuclear factor‐κB; p65, the RelA subunit of NF‐κB; ROS, reactive oxygen species; SASP, senescence‐associated secretory phenotype; SIRT1, sirtuin 1.

### 6.2. Animal Models of Atherosclerosis

Genetically engineered mouse models remain the cornerstone of preclinical atherosclerosis research. The apolipoprotein E‐deficient (ApoE^−/−^) and the LDLR^−/−^ mice are the most widely utilized. When fed a high‐fat diet, these animals develop profound hypercholesterolemia and form spontaneous atherosclerotic lesions in their aorta and major arteries. These lesions recapitulate several key pathological features of human atherosclerosis, including intimal lipid accumulation, infiltration of inflammatory cells, formation of foam cells, and progressive plaque development. To specifically study senescence within this context, more advanced models have been generated. These incorporate additional genetic modifications, such as fluorescent reporters or inducible suicide genes under the control of senescence‐specific promoters like p16^INK4a^, enabling the precise tracking or selective ablation of senescent cells within plaques [7, 101].

Senotherapeutic interventions in these animal models are usually delivered through dietary supplementation or direct pharmacological delivery (e.g., oral gavage, intraperitoneal injection) during treatment durations of several weeks to months. The effectiveness of these interventions is assessed by means of comprehensive plaque examination. This is accomplished by quantitatively evaluating lesion areas in the cross‐sections of the aortic root and the entire aorta on an en face preparation. The amounts of specific cell types (macrophages, smooth muscle cells) and matrix components (collagen) are evaluated using immunohistochemistry for compositional analysis. Measures of key morphological characteristics related to plaque vulnerability, namely, necrotic core size and fibrous‐cap thickness, are also taken. Because lesions in these models seldom rupture spontaneously, such measures are surrogate indices of cap stability rather than evidence that rupture is prevented. Molecularly, tissue samples are examined for changes in the expression of senescence markers (p16, p21), inflammatory cytokines, MMPs, and markers of oxidative stress [11, 20].

### 6.3. Representative Preclinical Findings With Natural Senotherapeutics

There have been promising results from the use of TMP in studying atherosclerosis in rabbits. These studies document that there is a dose‐dependent effect of decreasing atherosclerotic plaque area, with efficacy that varies between about 30% and 60% relative to controls treated with the vehicle. Other advantages observed are reduced lesion thickness and monocyte infiltration in the arterial wall. TMP therapy is also associated with a positive effect on systemic lipid profiles, which entails the reduction of total cholesterol, LDL cholesterol, and triglyceride levels and the elevation of HDL cholesterol levels. In vivo studies using mechanistic analyses indicate a concomitant decrease in vascular adhesion molecule expression, proinflammatory cytokine expression, and oxidative stress markers. In vitro confirmatory studies support these findings by showing that TMP mitigates the harmful effects of ox‐LDL, protecting endothelial cells and thereby maintaining endothelial function and preventing inflammatory stimulation [37].

Studies on flavonoids such as quercetin and fisetin in ApoE−/− mouse models have shown similar antiatherosclerotic effects. The senolytic combination of dasatinib and quercetin (D + Q), by contrast, has not been shown to reduce lesion burden. In Western diet‐fed ApoE−/− mice with established disease, intermittent weekly D + Q for 2 months cleared telomere‐associated foci‐positive senescent cells from the aortic media but not from within intimal plaques, and left intimal plaque size, plaque lipid content, macrophage (F4/80) burden, and plaque fibrosis were unchanged; endothelium‐dependent relaxation to acetylcholine was likewise not improved in these atherosclerotic animals, and carotid cross‐sectional compliance and distensibility were unaffected. The benefits that were observed were confined to improved relaxation to nitric oxide donors, increased phosphorylation of eNOS and VASP, and a significant reduction in intimal plaque calcification with corresponding loss of Osterix expression—a change notable because calcified lesions are typically refractory to lipid‐lowering therapy and because it occurred without any change in plasma cholesterol [20]. No published D + Q study in ApoE−/− or LDLR−/− mice has reported a reduction in lesion area, and circulating or intraplaque SASP factor levels have not been systematically quantified in these models; the present evidence therefore supports an effect of D + Q on vasomotor function and plaque calcification rather than on plaque size or composition.

Research on resveratrol in hyperlipidemic animal models indicates that its supplementation can attenuate atherosclerotic lesion development [70]. These benefits are accompanied by improvements in endothelial function and a reduction in systemic oxidative stress. The activation of SIRT1 appears to be a central mechanism for resveratrol’s effects, as interventions that genetically or pharmacologically inhibit SIRT1 abolish its protective actions [75, 78]. Curcumin interventions also reduce plaque burden in a dose‐dependent manner, coupled with a reduction in inflammatory markers within lesions [79, 80]. Combination approaches using two or more natural compounds have so far been examined mainly in cell‐based systems rather than in atherosclerotic animals. In human endothelial cells first confirmed to be senescent, a combination of resveratrol, curcumin, and β‐caryophyllene lowered secreted IL‐1β and IL‐6 at concentrations at which the single compounds were ineffective, although that effect was confined to the replicatively senescent model [76]. Whether such combinations reduce plaque burden in vivo more than the single agents has not been tested. The contrast between a vulnerable plaque and the stabilized plaque that senotherapeutic intervention is intended to produce is shown schematically in Figure 4.

**FIGURE 4:**
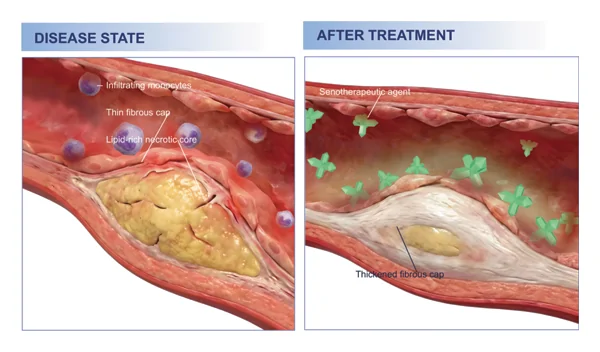
Schematic comparison of a vulnerable atherosclerotic plaque before and after senotherapeutic intervention.

Left panel, the untreated lesion: infiltrating monocytes cross the activated endothelium and accumulate in the intima, a lipid‐rich necrotic core occupies the center of the plaque, and a thin fibrous cap separates that core from the arterial lumen. Right panel, the same arterial segment after exposure to a senotherapeutic agent, meaning a senolytic or senomorphic compound directed at senescent vascular cells: Monocyte infiltration is reduced, and the lesion is covered by a thickened fibrous cap. The right panel shows the direction of change that senotherapy is intended to produce, plaque stabilization, and is not a composite of measured outcomes. As set out in Sections 6.3 and 6.4, cap thickening has not been demonstrated for every agent considered in this review; one synthetic senolytic reduced rather than increased fibrous‐cap thickness in mice with advanced lesions, lesion area was unchanged after treatment with dasatinib plus quercetin, and the murine lesions on which most of these data rest rarely rupture spontaneously. Both panels are schematic and are not drawn to scale.

### 6.4. Key Limitations and Translational Considerations

The interpretation and translation of findings from preclinical senotherapeutic studies necessitate a careful consideration of several important limitations. A primary challenge lies in the extrapolation of effective doses from rodent models to humans, especially for natural compounds with inherently poor bioavailability, where achievable tissue concentrations can differ markedly between species. Furthermore, significant heterogeneity exists across published studies in terms of experimental design, including variations in the doses used, treatment durations, the stage of disease at which intervention begins, and the methodologies employed for endpoint assessment. This heterogeneity makes it difficult to directly compare studies across different models and perform rigorous meta‐analyses. Another and more complex complication is the pleiotropic nature of most natural products; they tend to have a variety of biological activities other than senescence modulation (e.g., direct lipid‐lowering, antioxidant, or metabolic actions). This complicates the ability to clearly attribute any antiatherosclerotic effect seen solely to their senotherapeutic actions [34, 36].

The safety of chronic senotherapeutic interventions is also not well defined in the long term. Possible issues involve the potential for immunosuppressive conditions resulting from prolonged SASP suppression or simply the depletion of senescent cells, which could theoretically hinder host defense processes. What is also important to note is that not all senescent cells are deleterious; they have a physiological role in the body (e.g., wound healing, tissue repair, and suppressing cancer through oncogene‐induced senescence). Thus, any long‐term or nonselective senolytic treatment may have the potential to interfere with these beneficial processes, which could lead to delayed wound healing and an increased risk of cancer. In atherosclerosis, this concern is not merely theoretical: In Western diet‐fed ApoE−/− mice with advanced lesions, the BCL‐2 family inhibitor ABT‐263 left lesion size unchanged but reduced smooth muscle cell content and α‐smooth muscle actin‐positive fibrous‐cap thickness while increasing endothelial‐to‐mesenchymal transition, and was associated with a mortality rate above 50%, so senolytic treatment of established plaque cannot be assumed to be safe [102]. Lastly, one should remember that even the most suitable mouse models used to study atherosclerosis are not perfect. Differences in lipoprotein metabolism, immune responses, and plaque composition limit the direct predictability of these models for human clinical outcomes; in particular, the lesions they form rarely undergo the spontaneous fibrous‐cap rupture and occlusive thrombosis that underlie acute cardiovascular events in patients. These collective considerations powerfully underscore the imperative for well‐designed human clinical trials to validate the safety and efficacy of senotherapeutic approaches suggested by preclinical research [16, 103, 104].

Why, then, is it difficult to translate the preclinical promise of these compounds into demonstrated clinical cardiovascular benefit? Five conventional challenges apply. First, effective in vitro concentrations are rarely achievable in vivo: Culture work uses micromolar exposures, whereas the unmodified parent compounds reach only nanomolar plasma levels and circulate largely as Phase‐2 conjugates [78, 85], and a 2017 medicinal chemistry analysis called curcumin unstable, reactive, and nonbioavailable, with no double‐blind, placebo‐controlled trial then successful [105]. Second, lesion biology differs between species: The mechanism generating human events is not the one modified in mice [16]. Third, trials run for weeks to months against a disease of decades. Fourth, many trials assess surrogate endpoints, chiefly flow‐mediated dilation and biomarkers; improvements in these measures do not, by themselves, establish a reduction in cardiovascular events. The meta‐analysis of 884 randomized trials of 27 micronutrients reported heterogeneous clinical effects: n‐3 fatty acids reduced cardiovascular mortality, myocardial infarction, and coronary heart disease events; folic acid reduced stroke; coenzyme Q10 reduced all‐cause mortality in patients with heart failure; several supplements showed neutral effects on cardiovascular events; and beta‐carotene increased all‐cause mortality, cardiovascular mortality, and stroke risk [50]. Clinical event data were available for only eight micronutrients, so this analysis does not establish that surrogate improvements with curcumin or quercetin failed to translate into fewer cardiovascular events. Fifth, supplement‐grade material is inconsistent: Monacolin K varied more than 40‐fold between red yeast rice products with identical 600 mg labels [106]. We add a sixth, generated by this review. Applying the operational criteria of Section 5 showed that much of the striking preclinical literature was recorded in cells never confirmed to be senescent, so the senotherapeutic rationale has been carried forward on evidence that does not establish it. A trial built on that footing tests an unverified mechanism, and its failure indicts the compound, not senotherapy itself.

## 7. Omics Approaches to Elucidate Mechanisms and Identify Biomarkers

### 7.1. Transcriptomic Profiling of Senescence and SASP

The transcriptional landscape of senescence has been systematically mapped through bulk RNA sequencing analysis of senescent vascular cells. This analysis has revealed a coordinated upregulation of genes that encode SASP factors (cytokines, chemokines, and proteases), in addition to the anticipated downregulation of cell cycle progression genes and the upregulation of canonical senescence regulators (p16, p21, and p53). Single‐cell RNA sequencing (scRNA‐seq) has been implemented to analyze the heterogeneity of senescent populations. This enhanced resolution technique identifies distinct subpopulations of senescent cells that display distinctive transcriptional signatures and potentially divergent SASP profiles. Furthermore, the emerging technology of spatial transcriptomics allows for the precise anatomical localization of senescent cells, although to date it has been applied to atherosclerotic arteries in mice rather than to human plaque sections: combined single‐cell and spatial profiling of murine aortas has mapped senescent VSMC, fibroblast, and T‐cell populations during arterial remodeling and derived a vascular senescence signature [18]. The view that senescent cells are not randomly distributed but concentrate in regions of mechanical stress and vulnerability currently rests on histological and mechanistic studies of human plaques, including the demonstration that fibrous‐cap smooth muscle cells express SA‐β‐gal, p16, and p21 and carry markedly shortened telomeres, with telomere shortening tracking the severity of atherosclerosis [19], and the linkage of plaque‐cell senescence to intraplaque thrombosis by spatial multiomics of human and mouse lesions [107], rather than on spatially resolved transcriptomics of human lesions [11, 51].

The gene expression signatures derived from these transcriptomic studies serve dual purposes. First, they inform the development of molecular biomarkers capable of detecting and quantifying senescence burden in clinical samples. Second, they facilitate the identification of novel therapeutic targets within the SASP regulatory network. Comparative analyses of SASP profiles induced by different stressors (e.g., oxidative stress versus inflammatory cytokines) have delineated a common core of SASP components shared across contexts, as well as stressor‐specific factors [14, 108]. The application of these senescence markers to transcriptomic data from human atherosclerotic plaques has confirmed an enrichment of senescence‐related genes. Direct evidence that the degree of enrichment tracks with clinical severity or outcome is more limited: CDKN2A expression is higher in carotid plaque than in matched saphenous tissue and is associated with the severity of internal carotid artery stenosis and with symptomatic disease [109], and accelerated epigenetic age of the plaque predicts future cardiovascular events independently of blood epigenetic age and is associated with endothelial‐to‐mesenchymal transition [110]. Whether a transcriptomic senescence signature adds prognostic information beyond established clinical risk markers has not yet been formally evaluated.

### 7.2. Proteomic and Secretome Analysis

Mass spectrometry‐based proteomics provides an unbiased and comprehensive method for characterizing the SASP at the protein level, cataloguing hundreds to thousands of secreted proteins from the conditioned media of senescent cells. These studies have expanded the understanding of the SASP beyond the classic inflammatory cytokines to include a diverse array of molecules such as extracellular matrix proteins, growth and angiogenic factors, regulators of lipid metabolism, and components of extracellular vesicles (EVs). Quantitative proteomic approaches, which compare the secretomes of senescent versus nonsenescent cells or evaluate changes before and after senotherapeutic treatment, are instrumental in defining precise molecular signatures of the SASP and for quantifying the efficacy of interventions aimed at its modulation [14].

The analysis of EVs, including exosomes and microvesicles, represents a rapidly growing frontier in SASP research. Senescent cells release EVs with altered cargoes of proteins, lipids, and nucleic acids (e.g., miRNAs, fragmented DNA). These vesicles act as paracrine mediators that can transmit senescence to recipient cells, an effect demonstrated for small EVs acting through the interferon‐induced transmembrane protein IFITM3 [111]; their capacity to induce inflammatory activation in distant tissues and to contribute to the systemic effects of aging is inferred largely from in vitro and cell‐transplantation work rather than demonstrated in the vasculature. Due to their presence in accessible biofluids like blood, EVs are also being intensively investigated as a rich source of potential minimally invasive biomarkers for assessing systemic senescence burden [14].

### 7.3. Network Pharmacology and Target Prediction

The mechanism of action of natural products is typically polypharmacological, involving simultaneous modulation of multiple molecular targets rather than a single high‐affinity interaction. Network pharmacology is a computational systems biology approach designed to address this complexity [112]. It integrates diverse data types, including the chemical structure of compounds, predicted or experimentally validated protein targets, pathway databases, and disease association networks. This integration allows for the systematic mapping of the intricate relationships between a compound, its molecular targets, the biological pathways it affects, and the relevant disease phenotypes. For senotherapeutic natural products, network analyses frequently reveal a convergent perturbation of key nodes in the SASP regulatory network, such as NF‐κB, p38 MAPK, mTOR, and oxidative stress response pathways, while also forecasting potential off‐target effects and drug–drug interactions [113].

The true power of network pharmacology lies in its iterative nature. Predictions generated by computational models can guide the design of targeted experiments. The results from these experiments are then fed back to refine and improve the accuracy of the original models. This cyclical process accelerates the discovery of optimal therapeutic combinations, the identification of appropriate dosing regimens, and the stratification of patient populations most likely to respond to a specific intervention. Integrating these analyses with human genetic data, such as genome‐wide association study (GWAS) findings and Mendelian randomization analyses, can further strengthen the causal inference linking specific senescence pathways to atherosclerotic disease risk [114].

### 7.4. Biomarker Development for Personalized Approaches

The successful translation of senotherapeutics into clinical practice is heavily dependent on the development of reliable biomarkers. These biomarkers are needed to identify patients with a high burden of pathological senescence (patient stratification), to monitor the biological effects of treatment (target engagement and pharmacodynamics), and to predict individual therapeutic response. Circulating levels of certain SASP factors, such as IL‐6, IL‐1β, growth differentiation factor‐15 (GDF‐15), and PAI‐1, correlate with aging and cardiovascular risk and offer a starting point. However, their specificity is limited, as they can be produced by many cell types and processes unrelated to senescence. Therefore, current efforts are focused on developing multianalyte biomarker panels that combine measurements of specific cytokines, senescence‐associated metabolites, unique surface markers on circulating EVs, and signatures from cell‐free DNA. Such composite panels are intended to provide a more specific assessment of systemic senescence burden [115].

Beyond circulating biomarkers, advanced molecular imaging techniques are being explored. The development of positron emission tomography (PET) tracers that selectively bind to senescence‐associated targets, such as the enzymatic activity of SA‐β‐gal, could enable the noninvasive visualization, quantification, and spatial mapping of senescent cell burden within vascular tissues and other organs in living patients; such radioligands remain at an early stage of development, and although the β‐galactosidase‐targeted tracer [18F]FPyGal has shown selective accumulation in senescent cells in preclinical and early clinical studies, no senescence‐targeted tracer has yet been validated in the atherosclerotic arterial wall [116]. Combining such imaging data with circulating biomarker profiles and detailed clinical phenotyping would permit a multimodal assessment. In principle, such an integrated approach could support patient stratification and inform the choice between senolytic and senomorphic strategies [56], but this proposition has not been tested in any cardiovascular cohort.

These prospects must be set against five limitations of the circulating measurements now available. Sensitivity is low because senescent cells are rare and their secreted products are diluted in plasma, so a substantial vascular burden can coexist with unremarkable circulating values. Specificity, as noted above, is limited by nonsenescent cellular sources. Tissue origin cannot be resolved, since plasma IL‐6, GDF‐15, or PAI‐1 does not indicate whether the signal arose from the arterial wall, adipose tissue, liver, or bone marrow [117]. Standardization is lacking, as assay platforms, sample handling, and reporting units differ between laboratories, and no reference intervals have been agreed upon for these analytes [118]. Clinical cutoffs have accordingly not been established. The only analytically validated measurements remain the conventional cardiovascular risk markers, which do not report senescence burden, whereas SASP panels, EV signatures, cell‐free DNA, and senescence‐targeted tracers are investigational. No circulating senescence biomarker currently has an analytically validated assay or an established clinical decision threshold, so none can presently select between senolytic and senomorphic treatment.

## 8. Personalized Strategies and Clinical Translation

### 8.1. Heterogeneity in Senescence Burden and SASP Profiles

A fundamental consideration for clinical translation is the significant interindividual variability in senescence biology. Patients exhibit considerable differences in the rate of senescent cell accumulation, the cellular composition of senescent populations, and the qualitative and quantitative nature of their SASP. This heterogeneity is influenced by a complex interplay of factors, including genetic polymorphisms in genes governing senescence pathways (e.g., p53, p16), differential lifetime exposures to environmental stressors (e.g., smoking, pollution), the presence and severity of comorbid conditions (e.g., diabetes, chronic kidney disease, obesity), and lifestyle factors. Consequently, some individuals may develop a particularly high burden of senescent cells or a disproportionately proinflammatory SASP profile early in life, potentially identifying them as a subpopulation that would derive exceptional benefit from targeted senotherapeutic intervention [35, 119].

This inherent biological diversity argues strongly against a uniform, “one‐size‐fits‐all” therapeutic approach. A future paradigm will likely involve biomarker‐guided stratification. Patients identified as having a high total burden of senescent cells might be optimal candidates for senolytic therapies aimed at reducing this cellular load. Conversely, patients with a more moderate senescence burden but an exceptionally potent or damaging SASP might benefit more from a senomorphic strategy focused on silencing the harmful secretory output without eliminating the cells. The stage of atherosclerotic disease is another critical variable; interventions designed for primary prevention in individuals with early metabolic dysfunction may differ substantially from those intended to stabilize vulnerable, rupture‐prone plaques in patients with established cardiovascular disease [36]. The same logic is already being applied to biologic anti‐inflammatory therapy in atherosclerosis, where matching an agent to the inflammatory profile of the individual patient is proposed as the route to clinical benefit, and where immunosuppression, cost, and undefined long‐term safety argue against unselected use [29]. Whether senescence burden or SASP composition adds stratifying information beyond such inflammatory profiling has not been tested.

### 8.2. Dosing Strategies and Treatment Regimens

Determining the optimal dosing and administration schedules for senotherapeutics is an active area of investigation and may differ fundamentally between senolytic and senomorphic agents. For senolytics, a compelling strategy is intermittent or “pulse” dosing. This “hit‐and‐run” approach involves administering short courses of treatment to clear accumulated senescent cells, followed by extended drug‐free periods. This regimen capitalizes on the fact that senescent cells are largely postmitotic and are regenerated only slowly, potentially allowing for sustained therapeutic effects with minimal cumulative drug exposure and reduced risk of side effects. Preclinical studies suggest that such pulses, administered monthly or even quarterly, can maintain a reduced senescent cell burden, although the optimal frequency and duration for human applications remain to be defined [34, 120].

In contrast, senomorphic agents, which aim to suppress the continuous production of SASP factors, may necessitate more sustained or chronic administration to maintain effective pathway inhibition. However, continuous low‐dose regimens could potentially offer a favorable balance between efficacy and long‐term safety. An innovative approach under consideration involves combination strategies that pair intermittent senolytic pulses (to periodically reduce cell numbers) with continuous low‐dose senomorphic treatment (to suppress residual SASP secretion), potentially offering synergistic benefits while mitigating risks associated with either strategy used alone [121].

### 8.3. Integration With Standard Cardiovascular Therapies

Any novel senotherapeutic intervention will ultimately need to be integrated into the existing framework of standard cardiovascular care. This includes widely used medications such as statins, antiplatelet agents, antihypertensives, and newer drug classes like PCSK9 inhibitors, GLP‐1 receptor agonists, and SGLT2 inhibitors. A thorough evaluation of potential interactions is therefore essential, encompassing both direct pharmacokinetic drug–drug interactions and indirect mechanistic interactions. Interestingly, some established cardiovascular drugs, particularly statins, themselves possess pleiotropic anti‐inflammatory effects, including inhibition of NF‐κB signaling, raising the possibility of additive or synergistic benefits when combined with natural product senotherapeutics [16]. Under the operational criteria applied here, NF‐κB inhibition alone would not establish senomorphic activity, but direct evidence does exist for this drug class: simvastatin at 250 nM, a concentration within the serum range achieved during standard lipid lowering, reduced IL‐6 secretion by approximately 75% from human fibroblasts first confirmed to be senescent, leaving the growth arrest, the SA‐β‐gal‐positive fraction and persistent DNA damage foci intact [122], and a subsequent study reported reduced SASP and improved mitochondrial respiration in doxorubicin‐induced and replicatively senescent VSMCs [123]. Because statins may therefore act on the SASP through a pathway of their own, combination studies should carry senescence‐specific endpoints, so that additive, redundant, and antagonistic effects on the senescent cell burden and its secretome can be distinguished rather than assumed.

Clinical trial design must address key strategic questions. A primary question is whether senotherapeutics provide incremental clinical benefit when added to currently optimized guideline‐directed medical therapy. Alternatively, they may offer an alternative therapeutic avenue for patients who are intolerant or nonresponsive to standard treatments. The choice of endpoints in clinical trials is also critical. While traditional hard cardiovascular endpoints (myocardial infarction, stroke, cardiovascular death) are definitive, they require large, long, and expensive trials. The use of validated surrogate endpoints, such as imaging‐based measures of plaque burden or composition, vascular function tests, or specific circulating inflammatory biomarkers, could enable smaller, shorter, and more efficient proof‐of‐concept studies to establish biological efficacy and justify subsequent large‐scale outcome trials [28, 124].

### 8.4. Safety Considerations and Long‐Term Monitoring

Long‐term safety is a paramount concern for therapies that fundamentally alter a biological process as deeply embedded as cellular senescence. Senescent cells are not merely artifacts of aging; they serve important physiological roles in contexts such as wound healing, tissue repair following injury, and acting as a barrier against tumor development through oncogene‐induced senescence. Therefore, chronic and widespread senolytic therapy carries the theoretical risk of impairing these beneficial functions, potentially leading to delayed wound healing, compromised tissue regeneration, or an altered risk profile for certain cancers. Rigorous, long‐term surveillance for such potential adverse effects will be an essential component of clinical development programs [5, 103].

The immunological consequences of senotherapy also merit focused attention. While reducing chronic, sterile inflammation is a therapeutic goal, excessive suppression of the SASP or depletion of senescent immune cells might inadvertently compromise beneficial immune surveillance or host defense mechanisms against pathogens. This risk may be accentuated in the elderly target population, who already experience age‐related declines in immune function (immunosenescence). Thus, a key challenge will be to achieve a therapeutic balance that effectively quenches the pathogenic, chronic inflammation driven by senescence while preserving essential immune competence for protection and repair [104, 125, 126].

Natural product senotherapeutics generally benefit from a history of dietary consumption, which provides a baseline level of reassurance regarding acute toxicity. Compounds like quercetin and resveratrol have been consumed in foods for millennia. However, it is crucial to recognize that therapeutic supplements deliver these compounds at doses that far exceed normal dietary intake. Therefore, their safety profiles at these pharmacologically active doses cannot be assumed and require formal evaluation in well‐controlled clinical trials. Furthermore, the safety of combination regimens, which may be employed to enhance efficacy, needs systematic investigation to rule out unexpected interactions or cumulative toxicities [56, 57].

### 8.5. Economic and Regulatory Considerations

Cost and regulatory feasibility will decide whether senescence‐directed prevention reaches practice. Conventional cardiovascular risk stratification relies on inexpensive, universally available inputs: age, sex, blood pressure, a lipid panel, diabetes status, and smoking. Multiomics profiling of the kind described in Section 7 costs far more per person and requires specialized platforms and bioinformatic support unavailable in routine practice. To be fundable, a senescence‐directed test must add to those markers. Established criteria for novel cardiovascular risk markers require proof‐of‐concept, prospective validation in independent populations, incremental information beyond standard risk markers, an effect on management and outcomes, and finally cost‐effectiveness [127]. Most candidate senescence biomarkers have not cleared the second step, so trial enrichment and stratification of high‐risk subgroups, rather than population‐wide screening, are the realistic near‐term uses.

Their regulatory position is equally consequential. In the United States, quercetin, fisetin, resveratrol, and curcumin are sold as dietary supplements, which the Dietary Supplement Health and Education Act of 1994 placed under the framework for food rather than drugs: no premarket proof of efficacy, and no permitted claim to treat or prevent disease [128]. An atherosclerosis indication would require the drug framework. Botanicals also raise a manufacturing problem: extract composition varies with the raw material, its geographic origin, cultivation, harvest, and extraction process, and a global supply chain complicates quality assurance [129]. Chemical standardization, content uniformity testing, and batch‐to‐batch consistency are therefore prerequisites for interpretable trials, as implemented for grape polyphenol and resveratrol preparations supplied to a clinical study [130]. A dedicated botanical drug development pathway accommodates multicomponent mixtures, but its yield is low: more than 800 botanical investigational and preinvestigational submissions preceded 2018, yet only two botanical new drug applications had been approved [131].

Chronic primary prevention is the hardest setting in which to seek approval. Preventing a first cardiovascular event in asymptomatic people requires large samples, follow‐up measured in years, and hard clinical endpoints, since senescence burden and plaque composition remain investigational surrogates. The benefit‐risk threshold is also narrower, since years of treatment in healthy people tolerates little uncertainty about long‐term harm, including the risks to wound healing, tissue repair, and tumor suppression discussed above. These constraints, shared with other interventions directed at aging biology [132], favor initial development in patients with established disease and high senescence burden, where event rates are higher and trials smaller.

## 9. Conclusion

Cellular senescence and the SASP link the biology of aging to atherosclerotic cardiovascular disease. Senescent cells in the arterial wall are not inert: In experimental models, their sustained secretion of inflammatory cytokines, chemokines, and matrix‐degrading enzymes activates the endothelium, recruits immune cells, promotes foam cell formation, and erodes the fibrous cap. NF‐κB, p38 MAPK, mTOR, and persistent DDR signaling sustain this secretory phenotype and provide tractable targets for intervention.

Natural products offer several complementary entry points into this network. Quercetin and fisetin selectively clear senescent cells and are the only two compounds for which killing of confirmed senescent vascular cells has been shown against nonsenescent controls assayed in parallel [51, 65]. TMP, resveratrol, and curcumin have not been shown to suppress SASP output in confirmed senescent vascular cells: The effects attributed to them, namely, NF‐κB inhibition, lower ROS, reduced adhesion molecule expression, and preserved nitric oxide signaling, were recorded in cells that were never demonstrated to be senescent, and where resveratrol and curcumin have been tested directly against confirmed senescent human endothelial cells, neither reduced IL‐1β nor IL‐6 as a single agent [76]. In cellular and animal models, all five compounds have been reported to reduce lesion burden and improve plaque composition or endothelial function, although the evidence is uneven: a senescence‐specific endpoint has been measured alongside an atherosclerosis endpoint in the same animals for quercetin, for fisetin, and in a single report for resveratrol, whereas for TMP and curcumin no atherosclerosis experiment has included a senescence endpoint, and the reported benefits rest largely on broader anti‐inflammatory, antioxidant, lipid‐lowering, and vascular readouts.

Translating these results into clinical practice still requires resolving low bioavailability, defining optimal dosing schedules and treatment durations, characterizing long‐term safety, and identifying which patients will respond. Omics‐based profiling and circulating senescence biomarkers may close this gap by quantifying individual senescence burden, monitoring target engagement, and predicting therapeutic response.

Vascular senescence biology is now sufficiently developed to justify carefully designed early‐phase clinical studies, and biomarker, imaging, and analytical tools have advanced accordingly, although they remain investigational. The evidence summarized here is nonetheless predominantly mechanistic and hypothesis‐generating: Direct human cardiovascular interventional data are limited to small studies with surrogate vascular endpoints, and no trial has yet shown that a senotherapeutic modifies plaque progression or cardiovascular event rates. Senolytic trials in other age‐related conditions are already in progress, and their safety and efficacy data will inform the design of cardiovascular studies.

Realizing the therapeutic potential of SASP‐targeted therapy will require sustained collaboration across senescence biology, vascular pathophysiology, natural product pharmacology, clinical trial design, and regulatory science. Senescence‐directed therapy is unlikely to replace existing risk‐factor management, but it could become an additional pillar of precision cardiovascular medicine, one that targets the underlying biology of age‐related disease rather than its downstream consequences.

## Author Contributions

Dekun Liu and Sha Yang held the responsibility for the conceptualization and research design of the review. Napamanee Kornthong performed the literature search and revised the manuscript. Dekun Liu, Napamanee Kornthong, and Jakkree Tangthianchaichana handled the detailed data collection, execution of natural product‐based intervention analysis, and manuscript preparation. Shuguang Yu was in charge of reviewing the scientific data and providing resources. Dekun Liu and Prasert Sobhon performed the final data synthesis and critical analysis for the manuscript.

## Funding

This work was supported by the National Natural Science Foundation of China (No. 82374604) and the Central Guidance for Local Projects (No. 2024ZYD0129). This research also received financial support from the Thailand Science Research and Innovation Fundamental Fund, Fiscal Year 2025, and Research Unit in Innovative Marine Biotechnology and Natural Bio‐resources for Sustainable Health and Wellness, Thammasat University.

## Ethics Statement

The authors have nothing to report.

## Conflicts of Interest

The authors declare no conflicts of interest.

## Data Availability

No new data were created or analyzed in this study. Data sharing is not applicable to this article.
